# Genome of the green-head ant, *Rhytidoponera metallica*, reveals mechanisms of toxin evolution in a genetically hyper-diverse eusocial species

**DOI:** 10.1186/s13059-025-03777-2

**Published:** 2025-09-26

**Authors:** Anders Isaksen, Pedro G. Nachtigall, Robin A. Araya, Jia Hao Beh, Samuel D. Robinson, Thomas F. Hansen, Eivind A. B. Undheim

**Affiliations:** 1 Centre for Ecological and Evolutionary Synthesis, Department of Biosciences, University of Oslo, Oslo, Norway; 2 Institute for Molecular Biosciences, the University of Queensland, St Lucia, QLD Australia

**Keywords:** Venom, Gene evolution, Transposable elements, Toxin annotation

## Abstract

**Background:**

While ants are textbook examples of eusocial animals in which altruistic behavior is maintained through kin selection, several ants form genetically diverse colonies that challenge this concept. One example is the Australian green-head ant (*Rhytidoponera metallica*) whose colonies harbor such extreme genetic variation that they have been speculated to represent an unstable form of eusociality. Yet, *R. metallica* is among the most successful ants on the Australian subcontinent. This success has been hypothesized to be partly due to the diverse venoms harbored within each colony. However, the genomic basis and evolutionary scenarios that maintain this toxin diversity remain unknown.

**Results:**

To examine toxin genomic architecture, quantify individual-level genetic variation, and identify both proximate and ultimate mechanisms that have facilitated the toxin diversity in *R. metallica*, we generate a high-quality draft genome from a single worker. Most ectatotoxin genes are in clusters that contain evidence of multiple, complex gene-family expansions, some of which are likely explained by the presence of transposable elements. We also show that toxin regions of the genome exhibit elevated genetic variation despite being under strong selection and that this variation can translate to phenotypic diversity through toxin alleles with different functional properties.

**Conclusions:**

Taken together, our results point to classical gene duplication and diversification as the main evolutionary mechanism by which the main toxin family in ant venoms evolves, suggest toxin-gene functional diversification under frequency-dependent selection maintains colony-level venom hypervariability in *R. metallica*, and provide new insight into the role of multi-level selection in eusocial animals.

**Supplementary Information:**

The online version contains supplementary material available at 10.1186/s13059-025-03777-2.

## Background

Most ant species are usually thought to show reduced intracolony genetic variation due to their monogynous colony structure together with haplodiploid sex determination, in which female full sibs share on average ¾ of their genetic material [1–4]. However, deviations from the common monogynous colony structure are common in ants. Both multiple reproducing queens (polygyny) and multiple matings by a single queen (polyandry) are common in many ant species [1, 5–7], resulting in colonies of multiple matrilines and/or patrilines and workers with lower degrees of relatedness [8]. Another form of reproduction can also be found in some ant species where certain workers—so-called gamergates—mate and contribute to the reproduction of new progeny. Among gamergates, polygyny is more common than the conventional single-reproductive mating system [3, 9], challenging the fundamental idea that eusocial colony structure is maintained by kin selection based on close relatedness among nestmates [2].

Gamergate reproduction is mainly found in the subfamilies of Ponerinae and Ectatomminae [10, 11]. One extreme example is the Australian green-head ant, *Rhytidoponera metallica* (Smith, F., 1858, subfamily: Ectatomminae), in which as much as 5–15% of the colony can consist of gamergates [12]. When ready to mate, *R. metallica* gamergates emerge from the nest and release an attractant from their tergal gland to attract males from other nests [13, 14], suggesting that *R. metallica* gamergates are monandrous, i.e., mating with unrelated males from foreign nests [11, 15–17]. This reproductive strategy results in one of the lowest known degrees of intracolony relatedness in any ant species [18]. Due to this low intracolony relatedness, their social structure has been proposed to represent an unstable form of eusociality, and it has even been claimed that *R. metallica* is “degenerate and is probably headed for ultimate extinction” ([19], p. 220).

Despite the low relatedness within their colonies and proposed unstable form of eusociality, *R. metallica* is one of the most widespread and locally abundant ants on the Australian subcontinent [20–22]. Although low intracolony relatedness challenges the central idea of kin selection, genetic diversity may benefit group living [23, 24] by allowing for a wider range of niches to be exploited. More genetically diverse ant colonies have, for example, been observed to increase foraging performance [25], increase resistance against parasites and diseases [26–28], and increase brood production [29].

Another trait for which high intracolony variation could be advantageous is venom [30]. The majority of ants are venomous [31], where they produce biochemical cocktails of peptides and proteins that are injected into other organisms through a sting or by spraying into a bite wound. Since ant venoms can be a multifunctional trait harboring toxins that are used for defense, prey capture, and competition for territory and resources, especially with other ants [31–33], intracolony venom variation could provide a benefit to the colony in the form of a more diverse molecular toolkit. Although most ant species harbor less than 20 different peptide toxins colony-wide [34–38], *R. metallica* is one of the few exceptions, with colonies harboring up to or over 100 different ectatotoxins—short, predominantly linear peptide toxins with highly variable primary structures that dominate the venoms of ectatommine ants [30]. This number exceeds the number of peptide toxins fivefold compared to what is seen in other ant species (another exception being the ponerine ant, *Odontomachus haematodus* [39]), and led to the hypothesis that the low intracolony relatedness could itself be an adaptation for genetic diversity that enables *R. metallica* to thrive in such a wide range of habitats. However, although the venom diversity has been shown to be at least partly allelic [30], its genetic basis—and hence relation to intracolony genetic variation—remains unknown.

Here, we present a high-quality draft genome of *R. metallica* that was sequenced from a single worker, which allowed us to look specifically at the role of gene-family dynamics and the genetic variation harbored on an individual level. We first describe the genetic architecture of the ectatotoxin genes before we explore potential mechanisms responsible for the recent and likely ongoing expansion of the ectatotoxin gene family. Finally, we investigate levels of heterozygosity at the ectatotoxin regions and discuss different evolutionary scenarios that might explain the observed patterns of genetic variation and selection.

## Results

### Genome from a single ant resolves allelic and paralogous toxin diversity

Assembling the HiFi reads obtained from DNA isolated from a single worker of *R. metallica* (Additional file 1: Fig. S1) yielded a primary assembly with a total size of 395 Mb (394,639,337 bp) distributed across 507 contigs, which falls into the normal size range compared to other ant genomes [40] and fits well with the *k*-mer-based estimate of 372.1 Mb (Additional file 1: Fig. S2A). The assembly has high contiguity, with a maximum contig size of 11 Mb (11,586,707 bp), L50 value of 33 (number of ranked contigs containing half the assembly length), N50 value of ~ 3 Mb (3,430,620 bp), and N90 value of ~ 500 kb (494,692 bp). Genome-assembly completeness is 97.0% as measured against hymenopteran BUSCOs, of which 94.8% are complete single-copy BUSCOs and 2.2% are duplicated BUSCOs (Additional file 1: Fig. S2B). The final mitogenome assembly has a size of 17,121 bp, containing 37 identified genes in total, of which 13 are protein coding genes, 22 tRNA genes, and 2 rRNA genes (Additional file 1: Fig. S3). The mitogenome is distributed onto many different contigs of shorter size (Additional file 2: Table S1). As these did not interfere with the toxin-encoding regions, we did not remove these contigs from the final genome assembly before submission to NCBI.

Annotation using the funannotate pipeline resulted in identification of total of 31,255 genes. Although this overall gene annotation is relatively complete—as determined by measuring against both hymenopteran BUSCOs and non-ectatotoxin venom components—the annotated gene models included only nine ectatotoxin loci: three loci on contig 2 (two identical coding sequence regions of *Rm1a* and *Rm4a*), four loci on contig 7, and two loci on contig 27. These results highlight the difficulties in predicting genes encoding hypervariable short peptides such as ectatotoxins or other structurally similar short peptide toxins, which represent the main toxins of ants and other hymenopterans, and in *R. metallica* constitute almost 99% of the expressed venom [30]. We therefore annotated the 100 ectatotoxins identified in the colony venom gland transcriptome of *R. metallica* [30] by mapping their amino acid sequences onto the genome with both exonerate and miniprot. In addition, we mapped transcriptome reads from the same study and used ToxCodAn-Genome [41] for extra validation, supplemented by manual curation using FGENESH + (see methods). Using miniprot together with manual curation resulted in the annotation of a total of 45 ectatotoxin loci (Additional file 2: Table S2). The GC content within these ectatotoxin-encoding genes is approximately 46% compared to the overall GC content in the genome, which is 37.7%. Annotation of ectatotoxin genes using ToxCodAn-Genome specifically trained on ectatotoxins returned 32 of these 45 loci, suggesting toxin-specific gene predictors may be useful in identifying genes encoding short and hypervariable peptides.

In addition to ectatotoxins, we also identified several loci with genes encoding non-ectatotoxin venom proteins and peptides such as dipeptidyl peptidase 4 (DPP-4), phospholipase, CAP, crustacean neurohormone (CNH) and several precursory EGF-domain peptides previously described from the venom of *R. metallica* [30, 42]. Each of these venom components is located on different contigs from the ectatotoxins. The five EGF-like peptides identified from the pooled transcriptome data (ECTX_2_-Rm1a, ECTX_2_-Rm1b, ECTX_2_-Rm1c, ECTX_2_-Rm1d, ECTX_2_-Rm1e) are located on contig 4 and are encoded by one locus, suggesting that they are alleles. Dipeptidyl peptidase-4 (DPP-4) is also located at a single locus on contig 4, while the CNH is a single gene with three exons on contig 115. Although they can be detected in the venom, these non-ectatotoxin peptides and proteins are not major components and constitute less than 1.2% of the total expressed venom [30]. Hence, we focused on the ectatotoxins for all the subsequent analyses.

### Venom-encoding ectatotoxin diversity is encoded by a few clustered gene regions

**Fig. 1:**
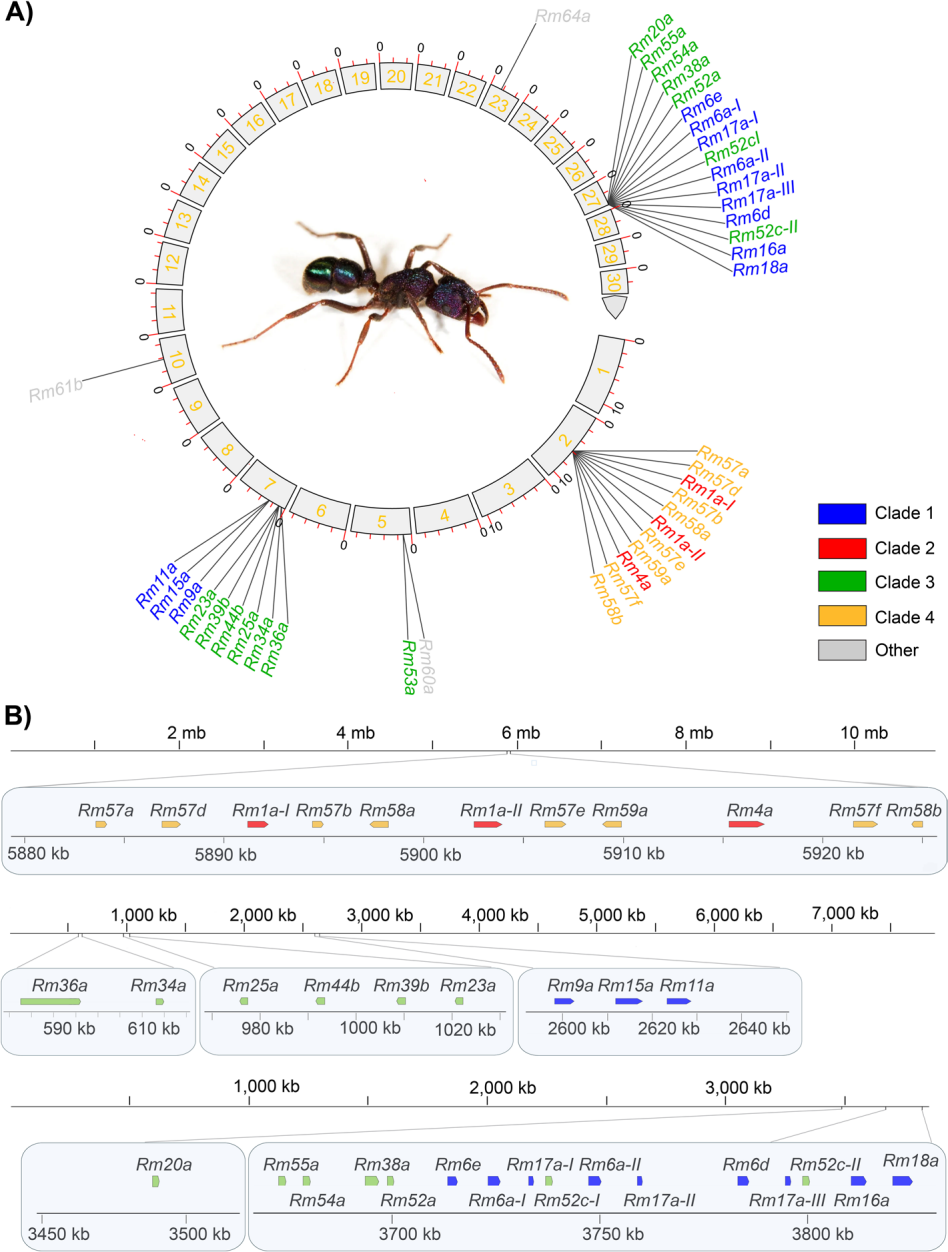
Toxin-encoding genes in the primary genome assembly of *R. metallica*. **A** Circular plot of the primary genome assembly showing the distribution of loci encoding ectatotoxins on the first 30 contigs, which correspond to almost half the assembly length. The components were identified from the pooled secreted venom of *R. metallica* [30]. **B** Locations of clustered ectatotoxin-encoding loci on contigs 2 (top), 7 (middle) and 27 (bottom). Contig 2 has one gene cluster containing ectatotoxins from clades 2 and 4. Contig 7 has three clusters, where each contains separate toxins from either clade 1 or clade 4. Contig 27 has one cluster plus one additional locus. The five clustered regions and the single locus on contig 27 contain 36 identified ectatotoxin-coding loci. The *Rm36a* locus on contig 7 likely represents two pseudogenized loci

Of the identified 45 ectatotoxin-coding loci, 36 are located within five different clustered gene regions distributed on mainly three contigs, namely contig 2, 7, and 27 (Fig. 1A, B): Contig 2 (~ 10.9 Mb) contains one ectatotoxin region in the middle of the contig (bp 5,883,552–5,925,068) that comprises eleven loci. Contig 7 (~ 7.9 Mb length) contains three ectatotoxin clusters, with 2, 4, and 3 loci for each region, respectively. These regions are located at the start of the contig in the forward orientation (total region bp 595,883–2,643,941). Contig 27 (~ 3.85 Mb length) contains one main gene cluster with 15 ectatotoxin-coding loci spanning bp 3,672,679–3,825,124. It also contains one single locus (Rm20a) downstream with location bp 3,488,477–3,490,334 (Fig. 1B). In addition to the 36 loci on contig 2, 7, and 27, additional loci are found on contigs 5 (2 loci), 10 and 23 (1 locus each), 35 (2 loci), 52 (1 locus), and 141 (2 loci). Previously, Robinson et al. [30] grouped the ectatotoxins (there referred to as aculeatoxins) into three different clades based on structural similarities: Rm1a–Rm5b constitute clade 2, Rm6a–Rm19a constitute clade 1, and Rm20a–Rm55b constitute clade 3. Here, we also define an additional fourth clade, which includes the ectatotoxins Rm57a–Rm61c (Additional file 1: Fig. S4).

**Fig. 2:**
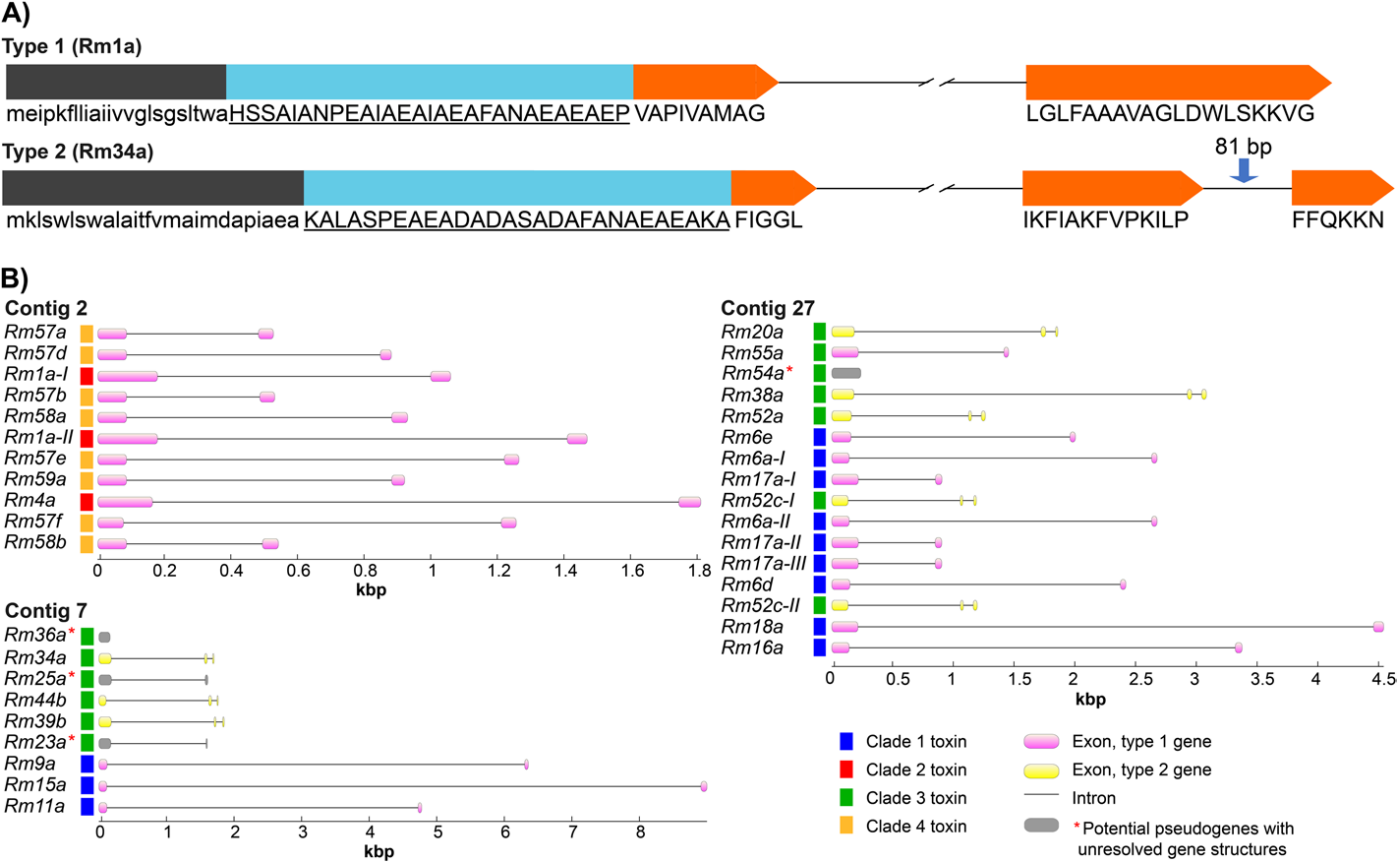
Ectatotoxin-encoding genes comprise two types of structures. **A** The type-1 ectatotoxin gene structure (top) consists of two exons, with the first containing signal- (black) and propeptide (cyan) as well as the first residues of the mature toxin (orange). In the type-2 ectatotoxin gene structure (bottom), the exon encoding the mature toxin peptide is split by a short intron. The length (81 bp) of the second intron is highly conserved among type-2 ectatotoxin genes, which belong mainly to clade 3 (located on both contig 7 and 27). Black and cyan regions correspond to signal- and propeptide, respectively, while the orange regions correspond to the mature part of the peptides. **B** Ectatotoxins, their respective clades and gene structures, and their distribution across the three contigs. Contig 2 consists only of type-1 ectatotoxin genes while contig 7 and 27 contain both types

The ectatotoxin-encoding genes have two main exon structures. The first type is characterized by two coding exons, in which the signal- and propeptide are coded by the first exon, and the mature peptide is coded by the last part of the first exon plus the entire second exon (Fig. 2A). Type 2 consists of three exons: Like type 1, the signal- and propeptide are coded by the first exon, while the mature part of the exon is coded by the last part of the first exon together with exons two and three (Fig. 2A). Of the 36 clustered ectatotoxin loci, 24 are of type 1—the most common type—while eight loci are of type 2, and four remain uncertain due to pseudogenization. Type 1 is found within all the clusters on all three contigs, while type 2 is mainly located on the largest cluster on contig 27, comprising five loci at contig 27 (*Rm20a*, *Rm38a*, *Rm52a*, *Rm52c-I*, *Rm52c-II*) and three loci on contig 7 (*Rm34a*, *Rm44b*, *Rm39b*) (Fig. 2B). Hence, contig 2 exclusively consists of type-1 ectatotoxin genes, while contig 7 and 27 contain both. All the type-2 gene structures are ectatotoxins that belong to clade 3, reflecting their close relatedness. However, on contig 27, all clade-3 ectatotoxin-encoding genes are of type-2 gene structure, except for *Rm55a*, which is the only clade-3 toxin with a type-1 gene structure annotated to contig 27.

**Fig. 3:**
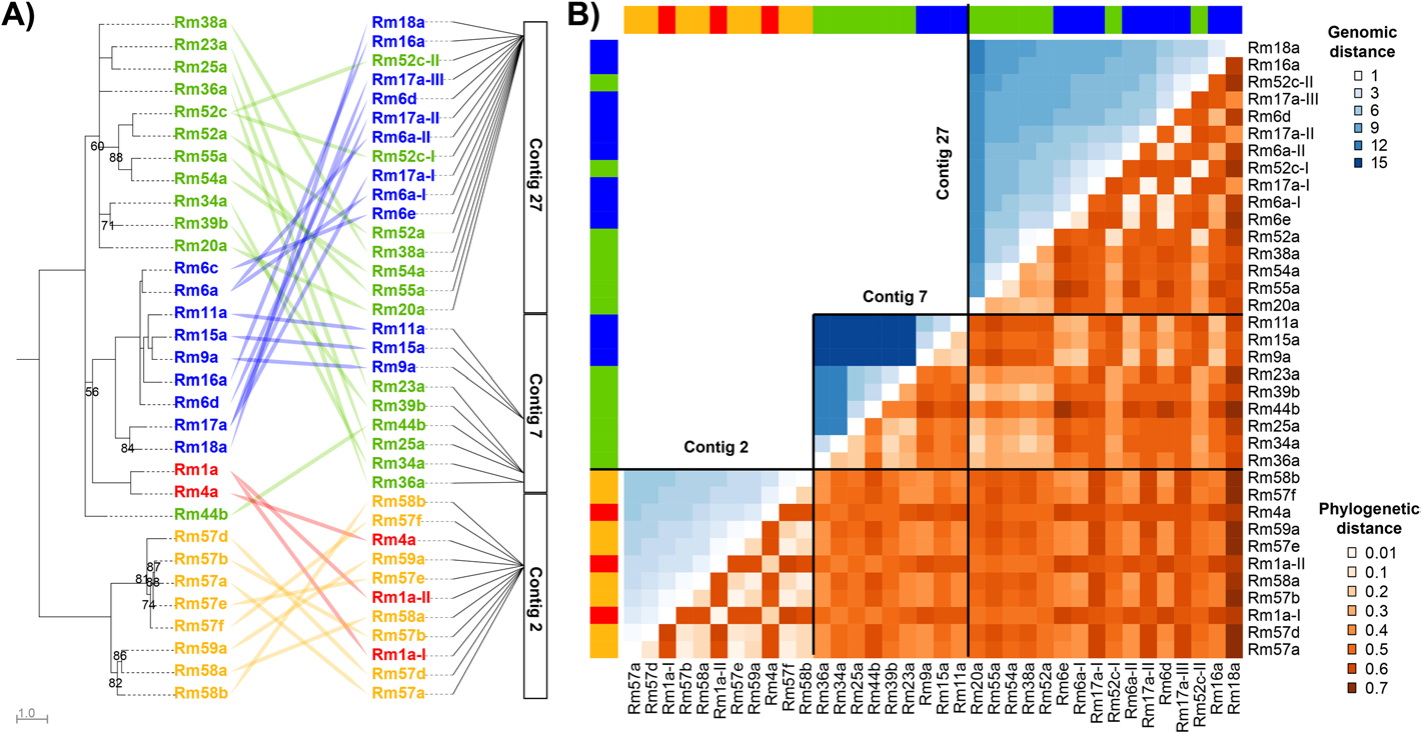
Ectatotoxin loci show a complex gene-family expansion pattern. **A** Ectatotoxin-locus phylogeny (left) is a poor predictor of physical location in the genome (right). Phylogeny is estimated by maximum likelihood under the VT + G4 model. Nodes with bootstrap support < 50% are collapsed, and only support values < 90% are shown. Colors correspond to clades 1–4 as per Fig. 1. Amino acid sequence alignment is provided as Additional file 3. **B** Heatmap of physical genomic distance (blue, above diagonal; log2-adjusted bp) and phylogenetic distance (red, below diagonal) illustrating pairwise comparison of all the ectatotoxin loci. Color bars on the left and on top indicate toxin clade (clade 1 = blue, clade 2 = red, clade 3 = green, clade 4 = orange). Contig 2 corresponds to the bottom left blue triangle, contig 7 to the middle blue triangle, and contig 27 to the upper blue triangle to the right. Pairwise comparison of genetic distances within each contig is shown in blue, while pairwise comparison of phylogenetic distances between the 36 ectatotoxin loci is shown in red. Physical genomic distances are measured in base pairs with log-adjusted values, with darker blue indicating greater distance. Phylogenetic distances are calculated as pairwise identities using nucleotide alignment of the mapped loci, with darker red indicating greater genetic distance. Nucleotide sequence alignment and distance tables are provided as Additional file 4 and Additional file 2: Table S3, respectively

The second intron in type-2 genes is both short (less than 100 nucleotides) and highly conserved with respect to sequence similarity. Five of the eight type-2 genes have second introns with a length of 81 bp, one gene has a second intron of 82 bp, there are two identical coding regions of *Rm52b* with 90 bp second introns, and *Rm39b* has a second intron of 98 bp. However, some of the most similar introns belonged to toxin genes that are not in genomic proximity. For example, *Rm20a*, *Rm44b*, and *Rm34a* cluster with respect to their second intron and the lowest pairwise similarity among these is 97.6%, with only two nucleotide substitutions. But while *Rm34a* and *Rm44b* are located on contig 7, *Rm20a* was found as a single locus on contig 27. Another example is *Rm52a* and *Rm38a*, which are in close genomic proximity to each other (2510 bp; Fig. 1B): Although their exon-based phylogenetic relationship suggests they are closely related (Fig. 3), their second introns are poorly conserved, with a pairwise identity of only 61.1%. These findings raise interesting questions about the evolution of the different gene structures, as closely related toxins neither necessarily have the same gene structure nor are found in close proximity (see also Fig. 3).

### Ectatotoxin genes show evidence of active gene-family expansions

Given the tandem repeats of ectatotoxins, we next examined whether dynamic and recent expansions by gene duplication could have contributed to these patterns (Fig. 1B). The ectatotoxin cluster on contig 2 includes two Rm1a-encoding loci with identical coding sequence exons but with different intron lengths (820 bp *versus* 1230 bp) due to repeated insertions of -GTGTGTGT- and -GTGCGTGC-. Besides these insertions, the introns are highly similar (97.7%), suggesting that the duplication of the locus encoding Rm1a might be a recent evolutionary event. Similarly, the ectatotoxin cluster on contig 27 also contains multiple identical coding sequence loci: Rm6a and Rm52c are encoded by two loci each while Rm17a is encoded by three loci. All the Rm6a- and Rm17a-encoding loci contain one intron (type-1 gene structure). In *Rm6a-I* and *Rm6a-II*, the introns are both 2488 bp and identical. Among the three Rm17a-coding loci, *Rm17a-I* has an intron length of 634 bp, while in *Rm17a-II* and *Rm17a-III* the intron length is 630 bp and 632 bp, respectively. *Rm17a-I* has an inserted -TATA-motif which is not present in *Rm17a-II*. In *Rm17a-III*, there is a deletion of -TA- with respect to *Rm17a-I*. In addition, the introns in *Rm17a-II* and *Rm17a-III* have two nucleotide substitutions. The duplicated Rm52c-encoding genes consist of two introns each (type-2 gene structure). The first intron differs in length between *Rm52c-I* (922 bp) and *Rm52c-II* (924 bp) and has a pairwise similarity of 96.5%, including several indels and substitutions. The second introns, which are much shorter (90 bp in *Rm52c-I* and 81 bp in *Rm52c-II*), have a pairwise similarity of 96.3%. This high similarity between the introns may indicate that expansions by tandem duplication of ectatotoxin genes have played an important, and ongoing, role in their evolution.

### Distributions of ectatotoxins show a complex phylogenetic/genomic relationship

To examine the patterns involved in ectatotoxin gene-family expansions in more detail, we compared the phylogenetic relationships of ectatotoxins with their physical distribution in the genome. This approach revealed that several clusters contain ectatotoxins from multiple clades and that their phylogenetic relationships are poor predictors of their genomic distributions (Fig. 3A). Of all clusters of ectatotoxin loci in *R. metallica*, only the three clustered regions in contig 7 consist of paralogs belonging to the same clades—the first two consist exclusively of toxins from clade 3, while the third region consists exclusively of toxins from clade 1. In contrast, the ectatotoxin cluster on contig 2 contains all toxins in clade 2 and clade 4 but interspersed with each other. Contig 27 also contains regions where two loci encoding clade 3 ectatotoxins are present between nine loci encoding clade 1 ectatotoxins. Thus, the clusters on contig 2 and 27 are mosaics of toxins from different clades with an overlapping tandem arrangement. Indeed, mapping the phylogenetic relationships against the genomic distance within clustered regions and contigs further highlighted several discrepancies between toxin distribution and phylogenetic relationship (Fig. 3B), suggesting complex family expansions have taken place.

### Toxin genes are associated with transposable elements that may have facilitated their functional diversification

**Fig. 4:**
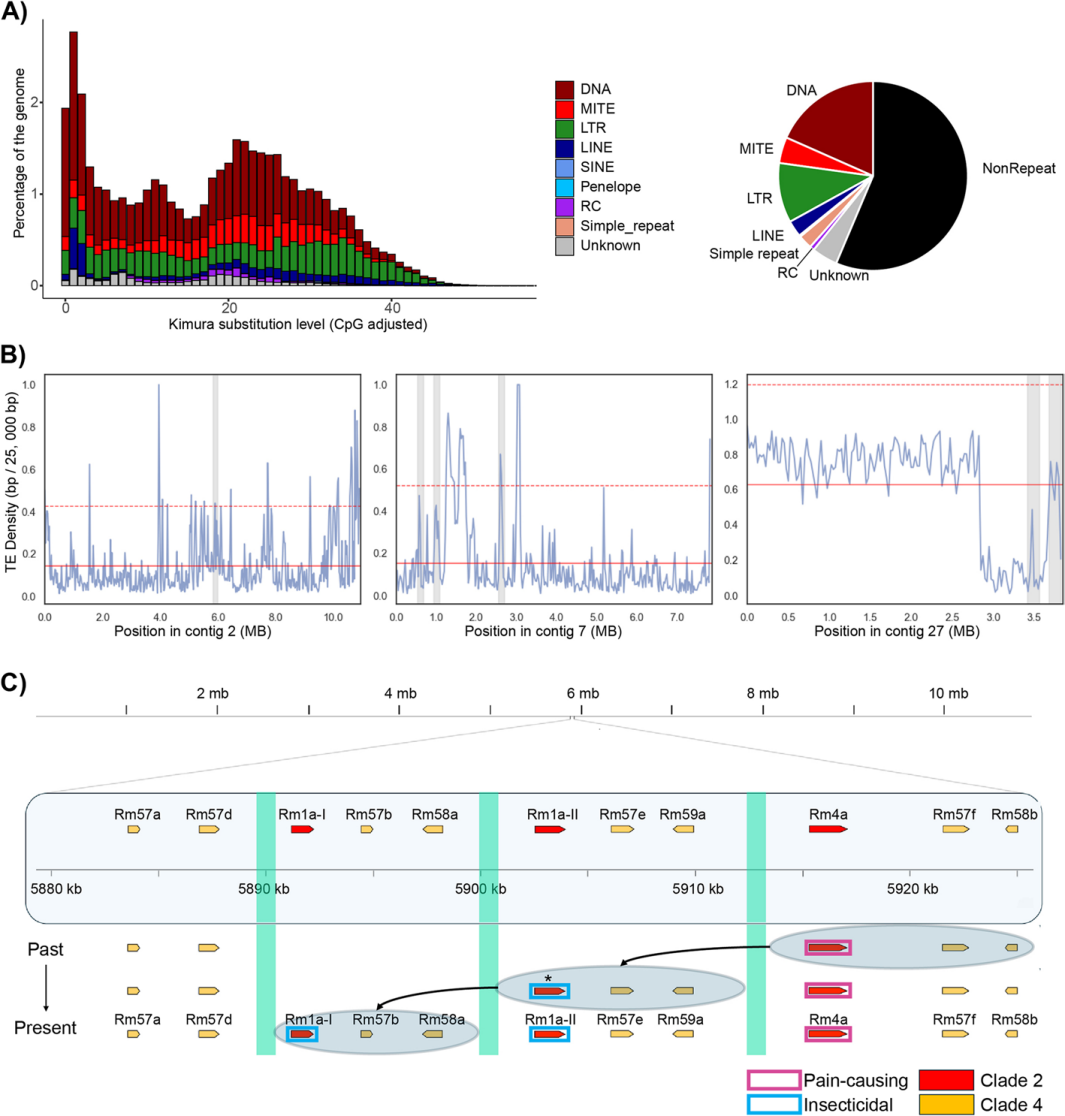
Ectatotoxin gene-family expansions may be facilitated by flanking transposable elements. **A** Repetitive landscape of the *R. metallica* genome, showing the amount and relative proportions of the different transposable elements. DNA repeats were the most abundant, followed by LTR, MITE, and LINE. The *y*-axis refers to the genome coverage for each type of transposable element. The *x*-axis shows calculated Kimura distances to their corresponding consensus sequence. Bars to the left do not diverge much from the consensus sequence and suggest recent copies, while bars to the right indicate more ancient copies. **B** Transposable-element density on contig 2 (left), contig 7 (middle), and contig 27 (right) was estimated in windows of 25,000 bp. Grey bars represent the ectatotoxin clusters, the solid red line represents the mean transposable-element density across the region, and the dotted red line represents the mean plus two standard deviations.** C** On contig 2, the transposable element Tc1/Mariner (turquoise bars) flanks *Rm1a* and *Rm4a*, suggesting a mechanistic hypothesis for the evolution of insecticidal *Rm1a* (blue boxes) derived from the defensive *Rm4a* (purple boxes). Ectatotoxins of unknown functions belonging to clade 4 are indicated by yellow boxes. The asterisk indicates neofunctionalization

The peculiar pattern of discrepancy between sequence similarities and physical genomic distances suggests that there are mechanisms that facilitate transpositions of genomic regions to create patterns of tandem duplications. With the hypothesis that repetitive elements might invoke such gene-family expansions, we annotated transposable elements and compared their distributions across the toxin and non-toxin regions of the genome. The repetitive landscape of the *R. metallica* genome consists mainly of class II DNA elements along with long terminal repeats (LTRs) and long interspersed nuclear elements (LINEs) (Fig. 4A). The repeat landscape also illustrates the accumulation history of transposable elements and suggests that there are many potentially recently active DNA transposons (Fig. 4A). We also found that all toxin regions contain a high density of transposable elements (Additional file 1: Fig. S5), and that the transposable-element density is higher in the toxin regions compared to the mean TE density of similarly sized windows of their respective contigs, particularly for contig 2 and contig 7, but less so for contig 27 (Fig. 4B).

As contig 2 comprises tandem-duplicated regions containing Rm1a- and Rm4a-encoding genes—which are thought to be large-effect loci with respect to pain-causing (defensive) and insecticidal (predatory) activity of the venom [30]—we focused on annotation of the different types of transposable elements surrounding the 11 loci on contig 2 (Additional file 1: Fig. S6–S8). The DNA transposon Tc1/Mariner was annotated to flanking regions of the two tandem-duplicated regions of *Rm1a-I*, *Rm1a-II*, and *Rm4a* (Fig. 4C, Additional file 1: Fig. S6). We also identified a DNA Sola transposon within the Tc1/Mariner region. However, it is likely that the Sola is a “false positive” integrated part of the Tc1/Mariner, and that Tc1/Mariner is the most Likely responsible transposable element for the tandem gene duplicated regions on contig 2 (Additional file 1: Fig. S9–S10). Considering the ectatotoxin phylogenetic relationships [30] and genetic distances (Fig. 3), we hypothesize that *Rm1a* evolved through a concerted duplication of *Rm4a* + *Rm57f* + *Rm58b*. We consider *Rm4a* the ancestral state due to the distribution of orthologs in many formicoid ants [43]. Following the functional diversification of the second copies into *Rm1a-II* + *Rm57e* + *Rm59a*, a recent second duplication of these two toxin-encoding genes resulted in a third set of paralogs containing an identical coding-sequence region of Rm1a (*Rm1a-I*) together with two new toxin-encoding genes *Rm57b* + *Rm58a* (Fig. 4C). This hypothesis is also supported by comparing the pairwise identities of *Rm57f*/*Rm57e* (97.6%), *Rm57e*/*Rm57b* (96.9%), and *Rm57f*/*Rm57b* (96.7%). Taken together, these findings suggest that the insecticidal Rm1a evolved from a vertebrate-specific defensive toxin (Rm4a) through gene duplications facilitated by Tc1/Mariner-type transposable elements.

Although Tc1/Mariner seems to be mediating tandem gene duplications of *Rm4a* + *Rm57b* + *Rm58b* on contig 2, similar transposable element annotations did not flank the tandem duplications of the genes encoding Rm17a, Rm6a, and Rm52c on contig 27. Thus, although transposable elements are associated with toxin-coding regions of the genome, we did not identify elements that are universally associated with, or potentially responsible for, all ectatotoxin gene clusters. Further studies are therefore needed to elucidate the evolutionary history and underlying genomic mechanisms of the tandem duplications on contig 27.

### Colony-wide venom composition contains functionally distinct ectatotoxin alleles

Although gene duplications explain some of the short venom peptide diversity observed within the colony of *R. metallica*, the number of ectatotoxin loci still accounted for less than half (45 of 100) of the ectatotoxins previously identified from pooled venom-gland transcriptome data [30]. Indeed, examining the degree of allelic variation in more detail by mapping these ectatotoxins to our primary assembly revealed that most loci have multiple allelic variants. In contig 2, five out of eleven loci (four out of ten non-identical coding regions) have multiple allelic variants, with the previously published pooled venomgland transcriptome (100 ants from the same colony) containing a total of 28 alleles across these loci (mean 4.5 alleles per variable locus). Similarly, the nine clustered ectatotoxin loci on contig 7 account for 35 alleles (mean 4.25 alleles per variable locus), while eight out of the sixteen loci in contig 27 (six out of the twelve non-identical coding regions) have a total of 33 alleles (mean 4.13 alleles per variable locus). Note that this allelic diversity is not restricted to ectatotoxins but is also present in the EGF-domain-containing toxins (ECTX_2_-Rm1a–e), where five homologs map to a single locus.

**Fig. 5:**
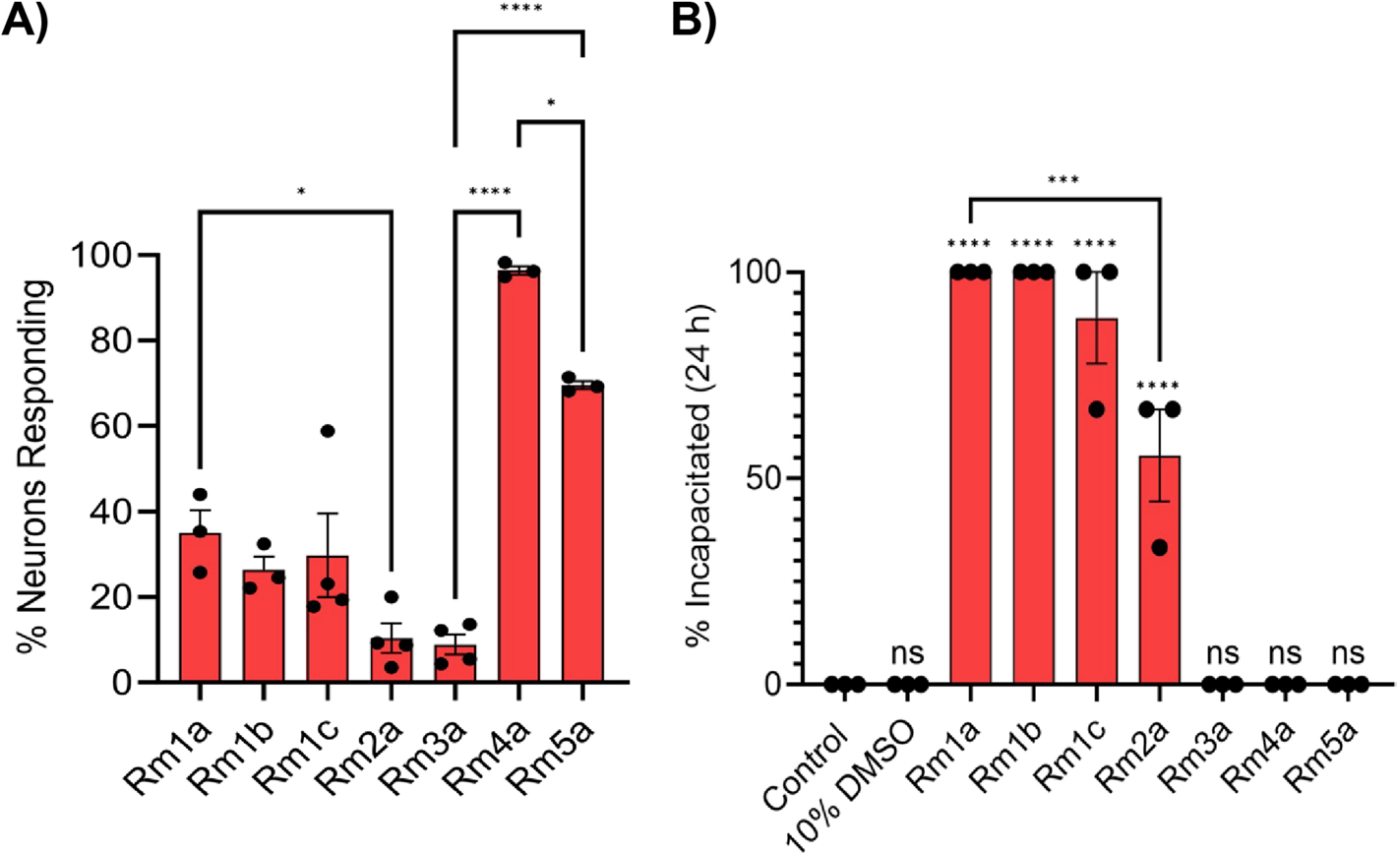
Ectatotoxin alleles exhibit different functional properties. **A** Effect of *R. metallica* peptide toxins on mouse dorsal root ganglion cells. Bars show the mean percentage of neurons that responded to 1 µM of each peptide across three independent experiments. Rm1a: mean = 35.1%, CI = 12.4–57.7%; Rm1b: mean = 26.4%, CI = 12.9–39.9%; Rm1c: mean = 29.8%, CI = − 1.2–60.8%; Rm2a: mean = 10.4%, CI = − 0.6–21.4%; Rm3a: mean = 8.9%, CI = 1.5–16.3%; Rm4a: mean = 96.4%, CI = 92.3–100.6%; Rm5a: mean = 70.0%, CI = 65.5–73.6%. CI refers to the 95% confidence interval. **B** Effect of *R. metallica* peptide toxins on house crickets (*Acheta domesticus*). Bars show the mean percentage of crickets incapacitated after intra-abdominal injection of each peptide (1.34 nmol/g; *n* = 3 independent experiments). Rm1a and Rm1b incapacitated all the crickets, while Rm1c and Rm2a incapacitated 88.9% (CI = 41.1–136.7%) and 55.6% (CI = 7.7–103.4%), respectively. CI refers to the 95% confidence interval. Statistical significance was tested using one-way ANOVA with Tukey’s multiple comparisons test and is indicated in **A** and **B** by asterisks: **** indicates *p* < 0.0001, *** indicates *p* < 0.001, * indicates *p* < 0.05. Non-significant *p*-values are indicated by ns. Significance above columns in **B** shows comparisons between each treatment and negative control.

In addition to having many allelic variants, there is a striking structural diversity among alleles mapping to each locus. Conducting a pairwise blast analysis on mature ectatotoxin amino acid sequences, we found no patterns of clustering within loci (Additional file 1: Fig. S11). This lack of clustering was also detected when projecting the embeddings of mature ectatotoxin peptides, suggesting substantial functional variation among allelic variants (Additional file 1: Fig. S12). To test the functional implications of this structural allelic variation, we synthesized and tested allelic variants of the two ectatotoxin loci with the clearest known toxicity phenotype. For the insecticidal Rm1a, we synthesized the alleles Rm1b, Rm1c, and Rm2a. For the vertebrate-specific pain-causing Rm4a, we synthesized and tested the alleles Rm3a and Rm5a. We found that 1 µM of Rm1a, Rm1b, Rm1c, Rm2a, and Rm3a all caused an activation of dorsal root ganglion (DRG) cells—which include pain-sensing neurons—defined as an increase in intracellular Ca^2+^ concentration, although slower and weaker than Rm4a and Rm5a, which activated 96% and 70% of the DRG cells, respectively. Rm1a activated on average 35% of the DRG cells, slightly higher than its corresponding alleles Rm1b (26%), Rm1c (30%) and Rm2a (10%) (Fig. 5A, Additional file 1: Fig. S13). The slow and weak activation of Rm3a (9% activation of DRGs) contrasts with the immediate activation caused by its allelic variants Rm4a and Rm5a (Fig. 5A, Additional file 1: Fig. S13N, Q; see also [30]). Rm5a activated DRG gradually, although slower than Rm4a (Additional file 1: Fig. S13Q, T). Injection into house crickets (*Acheta domesticus*) revealed that the insecticidal Rm1a and its allelic variants Rm1b, Rm1c, and Rm2a efficiently incapacitated the crickets. Rm1a and Rm1b incapacitated all the house crickets, while Rm1c incapacitated 89% of the house crickets on average (mean, *n* = 3). Rm2a was substantially less potent than the other three allelic variants, incapacitating 56% of the crickets on average (mean, *n* = 3). Rm3a, Rm4a, or Rm5a did not have any incapacitating effect (Fig. 5B). These results support the hypothesis that duplication followed by neofunctionalization plays a pivotal role in generating the diverse ectatotoxin arsenal that is present in colonies of *R. metallica* [30]. Different potency and potentially also function (e.g., Rm3a and Rm2a) further demonstrate that the allelic diversity contributes to an expanded functional toolkit on the colony level.

### Nonsynonymous heterozygous sites indicate elevated selection in toxin regions

**Fig. 6:**
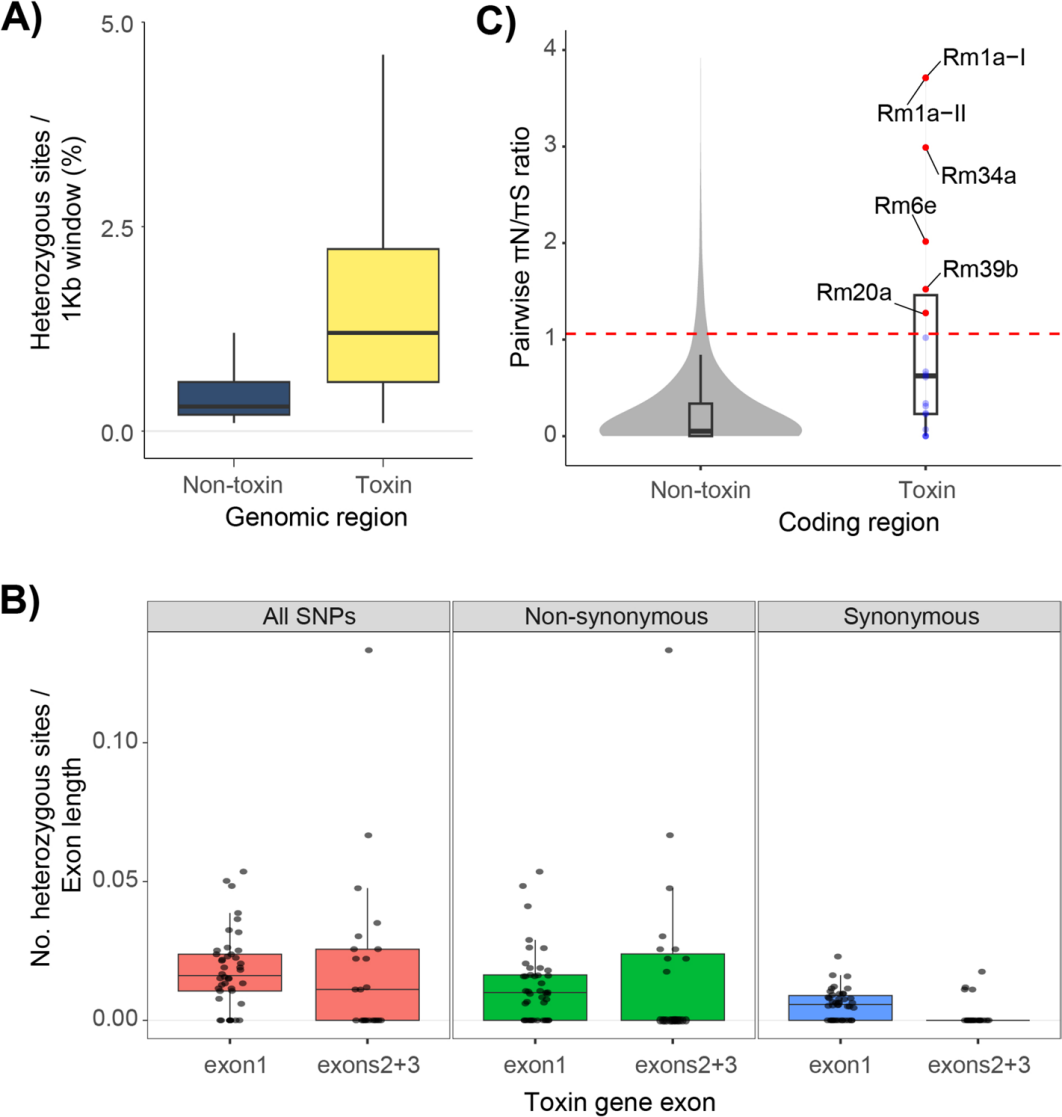
Toxin genes show elevated heterozygosity and high ratio of nonsynonymous heterozygotic nucleotides.** A** Toxin regions show higher levels of heterozygosity compared to non-toxin regions. Here, heterozygosity is defined as the number of heterozygous nucleotides divided by the total number of nucleotides in each window (toxins mean = 1.475%, non-toxin mean = 0.477%). Boxes show the interquartile range (Q1–Q3) and include the middle 50% of the data, in which the median (Q2) is marked with the horizontal line. **B** Total, nonsynonymous, and synonymous SNPs among toxin alleles in first exon (signal- and propeptide, *n* = 135) versus second and third exons (mature part of toxin, *n* = 77). First exon estimates also include loci of partially annotated genes where only the first exon was identified. Boxes show the interquartile range (Q1–Q3) and include the middle 50% of the data, in which the median (Q2) is marked with the horizontal line. **C** Pairwise π_N_/π_S_ ratio of heterozygote nucleotides in toxin and non-toxin coding genes with non-identical alleles. The red dashed Line represents the 95th percentile (= 1.0751) of π_N_/π_S_ distribution of non-toxin genes. Toxin genes exceeding the 95th percentile of the non-toxin genes are marked with red dots. A mean π_N_/π_S_ ≈1 for toxin genes fits the neutral prediction, while the mean π_N_/π_S_ of 0.248 for non-toxin genes indicates purifying selection

Given the combination of recent gene duplications and high intracolony allelic diversity among *R. metallica* ectatotoxins, we next examined whether this toxin diversity was also reflected in the level and types of heterozygosity. First, we used heterozygous pairwise single nucleotide polymorphisms to compare toxin to non-toxin regions throughout the genome. The heterozygous nucleotide variation per site (1-Kb window) was higher for the regions containing toxin genes, with a mean heterozygosity per site of 1.48% (interquartile range, i.e., Q1–Q3 (IQR) = 0.6–2.2%, median = 1.2%), compared to non-toxic regions in the genome, where the mean heterozygosity was 0.48% (IQR = 0.2–0.6%, median = 0.3%) (Fig. 6A). These results demonstrate that ectatotoxins are genetically diverse compared to the rest of the genome.

We further identified nonsynonymous and synonymous heterozygous nucleotides among the ectatotoxin exons. As the more conserved signal- and propeptide regions are coded by the first exon and the mature peptide is coded by the second and third exons, we expected to find a higher degree of nonsynonymous nucleotides in the second and third exons. Indeed, although we found a high number of nonsynonymous heterozygotic nucleotides at all exons, the second and third exons had a higher number of nonsynonymous heterozygotic nucleotides (IQR = 0–0.024 nucleotides per exon length) compared to the first exon (IQR = 0–0.016 nucleotides per exon length) (Fig. 6B). There were also more synonymous heterozygous sites on the first exon (IQR = 0–0.009 nucleotides per exon length) compared to the second and third exons, which have few synonymous heterozygotic nucleotides. This is what we would expect from conserved genic regions.

Finally, we estimated the rates of nonsynonymous to synonymous heterozygous sites (π_N_/π_S_) from pairwise comparisons of reference and alternate single-nucleotide polymorphisms. This approach revealed higher πN/πS ratios among toxin regions compared to non-toxin coding regions (toxin mean πN/πS = 0.95, IQR = 0.23–1.46, *n* = 48; non-toxin mean πN/πS = 0.25, IQR = 0.001–0.34, *n* = 9407) (Fig. 6C). Thus, while regions containing toxin genes have higher than expected heterozygosity and elevated nucleotide variation compared to the rest of the genome of *R. metallica*, a πN/πS ≈ 1 is a close fit to the neutral prediction. However, many of the ectatotoxins also have πN/πS > 1, such as *Rm1a* and its alleles, as well as *Rm34a*, *Rm6e*, and *Rm20a*, among others, suggesting these may be under strong positive selection (see Fig. 6C).

## Discussion

We generated a high-quality draft genome from a single worker of *R. metallica*, which allowed us to look specifically at the role of gene-family dynamics and the genetic variation harbored on an individual level. Ectatotoxins constituting the venom of *R. metallica* evolve primarily by tandem gene duplications, partially mediated by transposable element activity. Gene duplication followed by strong selection is often viewed as one of the major evolutionary processes for the evolution of animal venom toxins [44, 45], which are often described as evolving according to the gene birth-and-death model [46]. Indeed, we find both recent gene duplications, as evidenced by loci with identical and near-identical exons and introns, respectively, as well as at least four likely pseudogenized loci similar to toxins previously described from *R. metallica*.

The role of gene duplication has been questioned in some hymenopteran lineages, such as those from parasitoid wasps [47]. Indeed, although the ectatotoxin peptides have evolved through classical gene duplication and subsequent neofunctionalization, the larger venom proteins such as dipeptidyl peptidase 4 (DPP-4), phospholipase, CAP, crustacean neurohormone (CNH) and precursory EGF-domain peptides all occur as single-locus genes, with all variation observed in the venom being due to alleles. This exemplifies that different evolutionary mechanisms together work in concert to constitute the ‘venom cocktail’ in *R. metallica*. Indeed, Koludarov et al. [48] reported a dynamic interplay of different mechanisms of hymenopteran venom evolution, with some of the mechanisms being lineage specific. Interestingly, our findings in *R. metallica* with expansions of short linear toxins and fixation of larger proteins are the exact opposite of what is seen in, for example, bees, where melittin and melittin-like peptides, which are also short linear peptide toxins, tend to occur as single-copy genes while larger venom proteins occur as multiple paralogs [48]. Such observed contrasts within hymenopteran venoms highlight the complexity of their evolution and open for a plethora of interesting research questions in the future.

Although most ant species harbor less than 20 different peptide toxins colony wide [34–38], we identified 45 ectatotoxin loci. These results corroborate those from the only other detailed toxicogenomic analysis of an ant, of *Tetramorium bicarinatum* by Touchard et al. [49], where the genes encoding short linear peptide toxins called myrmicitoxins have undergone a similar gene-family expansion and are clustered in four main clusters of paralogs. There are also several likely orthologs with highly similar signal and propeptide regions and shared gene structures with *R. metallica* ectatotoxin genes. Among these orthologs, members of subfamily A2 in *T. bicarinatum* are particularly similar to clade 2 ectatotoxins in *R. metallica* (Additional file 1: Fig. S14), including Rm4a and U_3_-MYRTX-Tb1a (annotated as MYRTX_A2_-Tb3a by Touchard et al. [49]), which mature peptides share 66.7% similarity (74% across the full prepropeptide) and both likely play a defensive role by targeting vertebrate voltage-gated sodium channels [43]. However, while the A2 paralog cluster in *T. bicarinatum* consists of a tandem array of closely related sequences, the gene cluster containing clade 2 ectatotoxins is interspersed with genes encoding ectatotoxins in clade 4 (Fig. 1B, top). The putative orthologs to ectatotoxin clade 4 in *T. bicarinatum*, myrmicitoxin subfamily B1 (greatest pairwise similarity 81% across prepropeptides of MYRTX_B1_-Tb12a and Rm58a); however, form a tandem array downstream of the A2 cluster. These differences indicate that the clade 2 and 4 paralog expansion in *R. metallica* and A2 and B1 expansions in *T. bicarinatum* occurred independently since the split between Ectatomminae and Myrmicinae. Further investigations are required to untangle the apparently dynamic gene-family evolution of short peptide toxins in ants, including the likely loss of toxin paralogs in some species and complete loss of venom in others.

Given the frequent, and recent, toxin gene duplications in *R. metallica*, we next examined whether the duplications were associated with transposable elements, which are often involved with generation of new structural genomic variation. Indeed, we found that transposable elements were enriched in regions containing ectatotoxin genes, particularly for DNA transposons of the mariner superfamily. Ant genomes are known to have several mariner transposable elements [50], which are well known for their ability to be inserted into new regions of the genome by a “cut and paste” mechanism, through which new insertions often occur in proximity to the initial transposon [51]. Such processes can transpose genomic regions to new areas and corroborate well with the toxicogenomic landscape that we observe in *R. metallica*. Although the current role of transposable elements on contig 7 and contig 27 remains unclear, ancestral transposable element activity along with conserved duplications on contig 27 may explain the complex toxicogenomic landscape in these regions as well. Given the potent nociceptive activity of Rm4a against mammals and the insecticidal activity of Rm1a—but not vice versa [30]—our hypothetical evolutionary scenario in contig 2 suggests that the defensive Rm4a gave rise to the insecticidal Rm1a through ongoing, transposable-element-mediated gene-duplication events. This result also fits well with the hypothesis that ant venoms initially played a defensive role and later on evolved toxins with other functions used in prey capture, among others [52].

Although transposable elements activity partially explains the observed gene expansion, the reproductive nature of *R. metallica* also allows us to speculate how such a diversity comes into play in the first place. In addition to the low genetic relatedness within colonies, *R. metallica* is also considered a species complex [53]. Given the sexual calling behaviour of gamergates, which could attract males from closely related species with overlapping pheromones, it is plausible that hybridization events and subsequent introgression could take place and generate the extraordinary genetic diversity. Identifying whether hybridization and introgression do occur would require more population genomic data from additional colonies, but it is interesting to note that karyotype numbers across and within populations differ in *R. metallica* [16, 54]. However, the elevated heterozygosity and high proportion of nonsynonymous heterozygotic nucleotides in the ectatotoxin genes compared to non-toxin regions suggest that potential hybridization and introgression events alone cannot explain the observed differences in allelic diversity across the genome. Although the cause(s) remain to be elucidated, we evaluate four potential hypotheses to explain these patterns.

### Higher mutation rates in toxin-coding regions

Higher levels of heterozygosity in the toxin regions could reflect a higher mutation rate in these regions of the genome, which could compensate for the loss of genetic variation due to directional selection. It is interesting to note that higher GC content—which is what we find in the toxin regions compared to the non-toxin regions—is associated with greater mutation rates of both single-base substitutions and indels in yeast [55]. Although the transferability of these findings remains uncertain, the high GC content of the *R. metallica* toxin-encoding genes could provide one mechanism by which high genetic variation is maintained through focal mutagenesis in the toxin regions. We still find it unlikely that an increase in GC content from 37.7% in non-toxin regions to 46% in the toxin regions is sufficient to explain a threefold increase in heterozygosity (from 0,48% to 1,48%, see Fig. 6A). Greater sample size and population-wide evidence would be required to assess the significance of these elevated GC levels in the toxin regions and their potential role for ectatotoxin evolvability.

### Weaker selection on ectatotoxins

Another possibility is that the toxin region can be under weaker selection than the non-toxin region, as indicated by the mean π_N_/π_S_ ratios for the toxin regions (which fits the neutral prediction) compared to the non-toxin genes (which appear to be under purifying selection). For example, there might be no selection on toxin genes in haploid males, which lack a sting. Neutral or less beneficial alleles could potentially be maintained within colonies in highly polygynous colonies where each gamergate mates with different unrelated males. Still, we find it unlikely that these ectatotoxins evolve neutrally: although the mean π_N_/π_S_ ratio of ectatotoxins fits the neutral prediction, there is large variation in individual ratios with several ectatotoxins having ratios substantially greater than 1 (see Fig. 6C). For example, the two identical Rm1a-encoding paralogs have one of the highest π_N_/π_S_ ratios, suggesting that they are under strong positive selection. *Rm1a-I* and *-II* are the products of a recent duplication from a neofunctionalized paralog of *Rm4a*. We therefore interpret this as a plausible example of positive selection driving the evolution of an insecticidal toxin from a vertebrate-specific ancestor, rather than an example of neutral evolution. It should be emphasized that the π_N_/π_S_ ratios are not direct measures of selection as the data here are obtained from a single genome. Nevertheless, the difference in π_N_/π_S_ ratios still indicates variation in nonsynonymous nucleotides at the different loci, suggesting that not all ectatotoxin loci, if any at all, are experiencing weak selection.

### Ongoing selective sweeps

Although selective sweeps reduce genetic variation due to hitchhiking of neutrally linked sites, there could still be considerable variation at the onset of a selective sweep after a recently introduced beneficial mutation. We describe five major ectatotoxin regions (Fig. 1A, B), which are unlikely to be linked with each other due to their distant genomic locations mediated by transposable element activity. In addition, approximately 50% of all the toxin regions show higher heterozygosity levels compared to non-toxin regions (Fig. 6A), implying that the variation is much higher than what we would expect during selective sweeps. We also find little evidence of any fixed alleles and linked loci in the previously published mass spectrometry data of venoms milked from individuals from the same colony, where no ants appear to share the same combination of toxins [30]. This observation suggests that there may be high recombination rates also within ectatotoxin gene clusters, although further data are required to confirm this.

In addition to the 45 different ectatotoxin genes coding for the 100 identified ectatotoxins, there were several toxins that were not accounted for by the transcriptomes that are clearly present at the colony level. This observation suggests that there might be an even higher degree of intracolony variation among the workers than previously reported [30]. The total number of ectatotoxin loci, together with allelic variants for many of the loci, generates a myriad of potential combinations of expressed toxins constituting each individual’s venom profile, and the ectatotoxin combinations of individuals within colonies appear more or less unique [30]. Together, these findings do not fit with the patterns of selective sweeps.

### Frequency-dependent selection

A fourth hypothesis, which we find the most likely, is that the toxin regions are under some pattern of frequency-dependent selection. Strong directional selection would lead to fixation of alleles with beneficial mutations and lead to reduced levels of heterozygosity and nonsynonymous nucleotide variation, which contradicts our findings of elevated heterozygosity in toxin regions, including the presence of functionally variable and less potent alleles (Fig. 5). On the other hand, genetic diversity can be maintained in populations by heterozygote advantage or negative-frequency-dependent selection. Although any individual benefits of being a bearer of heterozygote toxin combinations are unclear, we suggest that the signs of selection in the toxin genes reflect frequency-dependent selection at the colony level maintaining rare alleles. *R. metallica* is a generalist, preying on a wide range of different arthropods and invertebrates [56]. It is possible that unique toxin combinations among workers enable the colony as a superorganism to exploit more resources and more accurately target a wide range of different prey and predators. In addition, multifunctional toxins might also increase the defensive ability of the colony. Where an individual simply cannot express all toxin variants simultaneously, variation at the colony level enables the colony as a whole to express more toxin variants, which might increase the mutual benefits of different individual properties through “social heterosis” [57].

Thus, our results are consistent with maintenance of toxin variation and genetic variation through frequency-dependent selection at the colony level, enabling the colony to exploit a wide range of niches through different individual toxin properties. Although such frequency-dependent selection is less likely to occur in eusocial species with a single reproductive individual per colony, *R. metallica*—and other ants with similar colony structures—could provide an interesting exception due to its unusual reproductive system of numerous reproductive gamergates within single colonies. The ectatotoxins in *R. metallica* may therefore represent a peculiar case in which group selection for toxin diversity maintains the colony structure when the influence of kinship is diminished. Alternatively, group selection might favor genetic diversity and polygyny by gamergate reproduction, which will reduce the influence of kinship. This finding raises interesting questions about the levels and units of selection affecting the evolution of venom in genetically diverse ant colonies, and perhaps eusocial organisms in general.

## Conclusions

Although the venom of *R. metallica* is distinct from those of most other hymenopterans studied to date in terms of colony-level toxin diversity, its primary components—ectatotoxins—evolve according to a “classic toxin” gene evolutionary scenario of gene duplications and subsequent neofunctionalization that appears to be a common feature of ant ectatotoxins. These duplications are at least in part facilitated by the presence of transposable elements, resulting in clusters of ectatotoxin genes with complex evolutionary histories. Moreover, neofunctionalization appears to be driven by classical positive selection, although probably under a less typical frequency dependent selection regime. Although our genome from a single worker provides high-resolution insight into the genomic architecture of the toxin arsenal of *R. metallica*, several questions remain unanswered. These include the effect of GC-compositional bias on ectatotoxin mutation rates, the potential role of exon shuffling and other small-scale structural variation in increasing toxin genetic variation, and directly testing for selection through the determination of allele frequencies within colonies. To address these questions, additional sequencing of ectatotoxin gene regions among individuals within colonies should be performed, which is likely to provide further insights into the genomic mechanisms that influence the maintenance of eusociality.

## Methods

### DNA extraction and sequencing

To assess the genomic basis for toxin variation in *R. metallica*, we sequenced the genome of a single worker ant using PacBio High-Fidelity (HiFi) sequencing. We extracted DNA from one single worker of *R. metallica* collected at the University of Queensland Saint Lucia campus, Brisbane, Queensland, Australia. The ant was collected from the same colony used for the generation of proteotranscriptomic data by Robinson et al. [30], but approximately two years later. The worker was snap frozen in liquid nitrogen and kept at − 80 °C until DNA extraction. DNA extraction was carried out using the MagAttract® HMW DNA extraction kit for animal tissue (Qiagen) and the integrity of DNA assessed using a Fragment Analyzer (Agilent) (Additional file 1: Fig. S1A). 660 ng high-quality DNA was fragmented using Megaruptor3, and the resulting 289 ng DNA was used for library preparation with PacBio protocol for Preparing HiFi Libraries from Low DNA Input Using SMRTbell® Express Template Prep Kit 2.0, which included a nuclease treatment step. The final library (Additional file 1: Fig. S1B) was sequenced with approximately half a SMRT Cell 8 M on a PacBio Sequel II, yielding 295.1 Gb polymerase bases across 4,110,878 reads (Additional file 1: Fig. S1C). The raw reads were processed with the PacBio Circular Consensus Sequences pipeline to yield a total of 1,862,968 HiFi reads (Additional file 1: Fig. S1D). Measurement of DNA integrity, library preparation and sequencing were performed at the PacBio node of the Norwegian Sequencing Centre, University of Oslo Department of Biosciences.

### Genome assembly and annotation

To generate a high-quality draft genome, we assembled the resulting HiFi reads with Hifiasm v0.15.1–329 [58], using default settings. To estimate the genome size of *R. metallica*, we used Jellyfish v2.3 [59] to perform a *k*-mer count (*k* = 19) against the hifi reads. We then used R v4.4.1 to plot *k*-mer count distribution and estimate genome size based on the most likely homozygote coverage peak. Primary assembly contiguity was determined using Quast v5.2.0 [60], while its completeness was assessed by comparing against near-universal single-copy orthologs from “insecta_odb10” searching 1367 BUSCO groups using BUSCO v5.4.3 [61]. Contiguity, GC content, and completeness were visualized as a snailplot using blobtools v1.0 [62] (Additional file 1: Fig. S2). The assembly was then pre-processed by cleaning, masking, and sorting the contigs by size using the default settings of funannotate v1.8.13 pipeline [63] before it was annotated. Ab initio gene predictors were trained by “funannotate train” using the assembled, pre-processed *R. metallica* genome together with previously published venom-gland RNA-seq data (NCBI accession number: SRR13051311 [64]). Gene prediction was performed with “funannotate predict” using the trained ab initio predictors from “funannotate train.” We then used the same venom-gland RNA-seq reads to update gene models from the prediction using “funannotate update,” which relies on RNA-seq, Trinity, PASA, and Kallisto. Performing the same pipeline without including venom gland RNA-seq data and prt2genome function resulted in a complete absence of predicted ectatotoxin genes. For the functional annotation, we used the “funannotate annotation” command with input data generated from InterproScan v5.47–82.0 [65], eggNOGmapper v2.1.7 [66], and signalP v5.0 [67]. To assemble the mitochondrial genome (mitogenome) of *R. metallica*, we used the long-read mode of MITGARD [68] and set the mitogenome of *Wasmannia auropunctata* (NCBI accession number: NC_030541.1) as the reference. The resulting mitogenomic assembly was annotated using MitoZ [69], using “Arthropoda” as the target clade.

### Ectatotoxin annotation

Searching the known *R. metallica* ectatotoxins (NBCI accession number: MW317022-MW317128) against protein-coding genes predicted by the funannotate pipeline revealed that only 9 loci had been annotated, and we therefore annotated these separately. Because the “funannotate util prot2genome” function (using Diamond v2.0.15 [70] and Exonerate v2.4.0 [71] with max intron length set to 30,000 bp) mapped only some of the ectatotoxin-encoding genes, we also mapped ectatotoxin peptide amino-acid sequence to the genome with miniprot v0.7 [72]. Complementing this toxin annotation, we also used ToxCodAn-Genome [41] for comparison and validation. To predict exact locations of some of the unresolved and missing exons, we manually inspected expression data from the transcriptome and identified corresponding genomic regions using FGENESH + [73]. To generate an overview figure of the distribution of ectatotoxin loci across the assembly, we used TBtools-II v2.096 [74]. To classify ectatotoxins into phylogeny-based clades, we repeated the phylogenetic analysis by Robinson et al. [30]. Phylogenetic relationships were estimated by maximum likelihood with IQ-TREE v2.2.0 [75] based on Additional File 3 in Robinson et al. [30], using ModelFinder [76] to identify the best-fitting model and ultra-fast bootstrapping to calculate node support [77].

### Phylogenetic versus physical genomic relationships of ectatotoxins

To examine the relationship between relatedness and physical closeness of ectatotoxin loci in the genome, we estimated the phylogenetic relationships of ectatotoxin paralogs. We aligned amino-acid sequences using MAFFT v7.505 [78] and estimated their phylogenetic relationships by maximum likelihood with IQ-TREE as described above. We also calculated and compared their pairwise phylogenetic and physical distances. For the phylogenetic distance, we aligned ectatotoxin nucleotide sequences using MAFFT v7.505 and used it as input in the Biopython package [79] to calculate the phylogenetic distance based on identity. The physical distance between loci was calculated based on the distances in base pairs from the end of one gene to the start of another gene located in the same contig.

### Repetitive-region annotation

To identify repetitive regions and transposable elements (TEs) we followed the strategy previously described in Nachtigall et al. [80] and available in the following GitHub repository: https://github.com/pedronachtigall/Repeat-annotation-pipeline. We used RepeatModeler2 v2.0.1 [81] to generate a de novo species-specific repeat library, which was classified using RepeatClassifier. Transposable elements categorized as “Unknown” were classified using DeepTE v1.0 [82] with the model designed for metazoans. To remove false-positive repetitive elements, we filtered out any sequence classified as “NonTE” using TERL v1.0 [83]. We also used the repeat sequences available for twelve other Hymenoptera species (hereafter named as Hymenoptera TE library) obtained from a semi-curated transposable element library designed for several insect species [84]. For this library, we also classified transposable elements annotated as “Unknown” using DeepTE v1.0 and removed “NonTE” sequences using TER v1.0. Using the species-specific and Hymenoptera TE libraries, we then performed a serial repeat annotation using RepeatMasker v4.1.1 (https://www.repeatmasker.org/) through the following steps: (i) we annotated only simple repeats and used the masked genome as input to (ii) annotate the transposable elements using the Hymenoptera TE library. The masked genome was then (iii) annotated using only the “known” transposable elements classified by RepeatClassifier from the species-specific TE library; and finally, (iv) we used the reclassified “Unknown” transposable elements from the species-specific library to annotate the masked genome from the previous step. The annotations of all steps were then merged to generate the final repetitive annotation and masked genome. The divergence level between the individual transposable element copies versus their consensus sequences based on CpG adjusted Kimura distance was estimated using RepeatMasker built-in scripts. Furthermore, transposable elements that were located near ectatotoxins were manually curated using TE-Aid [85].

### Heterozygosity across the genome: toxin versus nontoxin regions

Given the high level of intracolony ectatotoxin variation, and the generally high genetic variance in the colonies of *R. metallica*, we examined the degree and distribution of heterozygosity across the genome. First, we mapped the HiFi reads against the assembled genome using pbmm2 (https://github.com/PacificBiosciences/pbmm2), which is a wrapper of Minimap2 [86] designed to use better parameters for mapping HiFi reads. Then, we used Samtools [87] to filter low-quality and multi-mapped reads by setting a mapping quality threshold to 30. The variant alleles were genotyped using the Genome Analysis Toolkit (GATK v4.2). We filtered VCF files to mask potentially erroneous genotype calls as recommended by GATK as follows: QD < 2.0, FS > 60.0, and MQ < 40.0. We kept only biallelic SNPs for downstream analysis. We calculated per-site heterozygosity in 1-Kb sliding windows across the genome of the sequenced individual as described by Stanhope et al. [88]. Here, heterozygosity is defined as the number of heterozygous nucleotides divided by the total number of nucleotides in each window (i.e., the denominator includes both variant and invariant positions). Then, we compared the windows containing and surrounding toxin genes (i.e., toxin genes plus 1-Kb upstream and downstream regions) against non-toxin regions to check for differences.

### Nonsynonymous and synonymous heterozygote nucleotides at ectatotoxin loci

To look for evidence of differences in focal heterozygosity at the ectatotoxin loci, we compared synonymous and nonsynonymous heterozygous nucleotides of the exons. We aligned the allelic sequences for each gene using the codon-based approach PRANK (v.170427). We also compared synonymous and nonsynonymous heterozygous nucleotides between toxin and non-toxin genes as described by Nachtigall et al. [89]. The codon-based alignments were used as input to estimate the pairwise synonymous heterozygote nucleotides (S), nonsynonymous heterozygote nucleotides (πN), and πN/πS ratios (πω) using codeml from paml package (v4.9). Genes with πS < 0.001 and πS > 0.10 were removed to eliminate putative erroneous gene annotations.

### Structural comparison of ectatotoxin allelic variants

To compare allelic toxin variants, we used CLANS [90] to cluster mature amino acid sequences of ectatotoxins based on all-against-all pairwise blastp E-values. We first used the CLANS web-utility (https://toolkit.tuebingen.mpg.de/tools/clans) to perform an all-against-all pairwise blastp analysis, using default parameters. We then used the Java-based CLANS tool to cluster and visualize the resulting similarity matrix, using P-values less than 1 and otherwise default parameters. To further explore structural and potential functional similarities, we generated embeddings for the same sequence dataset using the ProtT5-XL-u50 protein language model [91] as described in the repository and guide of bio embeddings (https://github.com/sacdallago/bio_embeddings) [92]. We then performed a principal component analysis using the sklearn package in Python that we visualized using the ggplot2 package in R.

### Peptide synthesis

All peptides were produced using Fmoc solid-phase synthesis at 0.1 mmol scale. Lys/Trp/His(Boc), Ser/Thr/Tyr(tBu), Asp/Glu(OtBu), Asn/Gln/His(Trt), and Arg(Pbf) were used as protecting groups. Peptides were assembled on Rink-amide ProTide resin (CEM, Matthews, NC) on a CEM Liberty Prime HT24 microwave synthesizer (CEM Corp) using *N*,*N*′-diisopropylcarbodiimide (DIC)/oxyma. Fmoc groups were removed with 20% pyrrolidine, as per manufacturer’s protocols. Peptides were released from resin by treatment with 95% TFA/2.5% H_2_O/2.5% triisopropyl silane. Peptides were precipitated with 15 mL ice-cold ether, extracted in A/B 50/50 (A: 0.05% TFA, B: 90% ACN, 0.045% TFA) and lyophilized prior to purification. Peptides were purified on a Shimadzu Prominence LC-20AT RP-HPLC system equipped with a SPD-20AV UV detector and a FRC-10A fraction collector using an Agilent C18 column (30 × 250 mm; particle size, 5 μm; pore size, 100 Å; Agilent Technologies, CA, USA) at 8 mL/min. Gradient used was 40–90% B over 50 min. Fractions of interest were lyophilized and purity assessed using ESI MS and analytical RP-HPLC. Stock solutions of Rm1b and Rm1c were prepared by dissolving lyophilized peptide in 100% dimethyl sulfoxide (DMSO) (1 mM final concentration). Stock solutions of Rm1a, Rm2a, Rm3a, Rm4a, and Rm5a were prepared by dissolving each lyophilized peptide first in 100% DMSO then diluting to 1 mM peptide, 5% DMSO (v/v) in H_2_O.

### Calcium imaging assay of mammalian sensory neurons

Dorsal root ganglion cells were isolated from 4- to 6-week-old male C57BL/6 mice purchased from the Animal Resources Centre (Australia). The cells were dissociated and then plated in Dulbecco’s modified Eagle’s medium (Gibco) containing 10% fetal bovine serum (FBS) (Assay Matrix) and penicillin/streptomycin (Gibco) on a 96-well poly-D-lysine-coated culture plate (Corning) and maintained overnight. Cells were loaded with Fluo-4 AM calcium indicator according to the manufacturer’s instructions (Thermo Fisher Scientific) at 37 °C for 30–45 min and then at room temperature for 45 min. After loading, the dye-containing solution was replaced with room temperature assay solution (0.1% BSA in Hanks’ balanced salt solution, 20 mM Hepes). Images were acquired at 10 × objective at one frame/s (excitation 485 nm, emission 521 nm). Fluorescence corresponding to intracellular calcium ion concentration, [Ca^2+^]_*i*_ of ~ 250 cells per experiment was monitored in parallel using a Nikon Ti-E deconvolution inverted microscope, equipped with a Lumencor Spectra LED Lightsource. Baseline fluorescence was monitored for 30s. At 30 s, assay solution was replaced with 100 µL assay solution (negative control), then at 1 min with 100 µL test peptide (1 µM in assay solution). Fluorescence was monitored for 2 min before test peptide was replaced with 100 µL KCL (30 mM; positive control). Experiments using mouse tissue were approved by UQ Animal Ethics Committee (2021/AE000812).

### Insect incapacitation assay

House crickets (*Acheta domesticus*; Pisces Live Food, QLD, Australia) (average mass 149 mg) were injected intra-abdominally with 2 µL of 100 µM synthetic peptide dissolved in water or 10% (v/v) DMSO (Rm1b, Rm1c, Rm5a) (1.34 nmol/g). Crickets were assessed for incapacitation 24 h after injection. Negative control crickets were injected with 2 µL of water or 10% DMSO. The percentage of crickets incapacitated by each treatment was compared by a one-way ANOVA with Tukey’s multiple comparisons test (GraphPad Prism 10.3.0).

## Supplementary Information

Additional File 1. PDF containing figures S1–S12

Additional file 2. Excel file containing tables S1–S3

Additional file 3. File with the aligned amino acid sequences of the coding regions of *R. metallica* ectatotoxin loci.

Additional file 4. File with the aligned nucleotide sequences of the coding regions of *R. metallica* ectatotoxin loci.

## Data Availability

HIFI reads and genome assembly are available under NCBI GenBank bioproject number PRJNA883125 and biosample number SAMN30959808 [93]. Gene annotations are available in the figshare database [94], while supporting data are included as additional data files. No custom code was used.
