# Supplementary material for: Genome of the green-head ant, Rhytidoponera metallica, reveals mechanisms of toxin evolution in a genetically hyper-diverse eusocial species
Source: Genome Biol. 2025 Sep 26;26:306. doi: 10.1186/s13059-025-03777-2 (PMC12465672; doi:10.1186/s13059-025-03777-2)
Supplement: Supplementary file 1 — Additional File 1. PDF containing figures S1–S12 [file 13059_2025_3777_MOESM1_ESM.pdf]

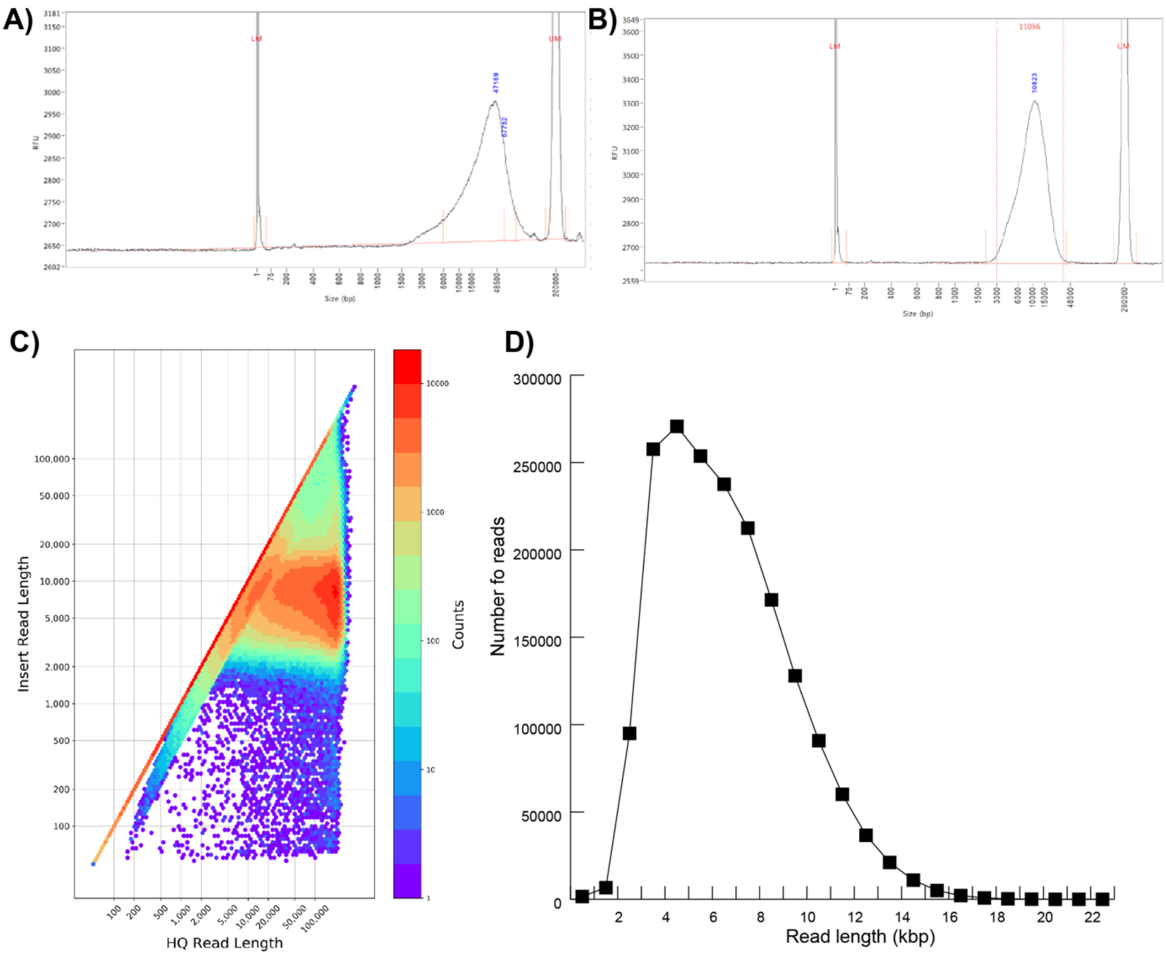

Fig. S1. Integrity of genomic DNA extracted from a single worker of *R. metallica*, resulting library used for genome sequencing, and read performance and hifi read length distributions. **A)** Genomic DNA extracted using the MagAttract HMW DNA kit (Qiagen). **B**) Size distribution of final library used for genome sequencing using half a SMRT Cell 8M on a PacBio Sequel II. and integrity measured using a Fragment Analyzer (Agilent). **C)** Plot showing insert length versus high-quality read length of raw reads. **D)** Length distribution of high-quality circular consensus (hifi) reads.

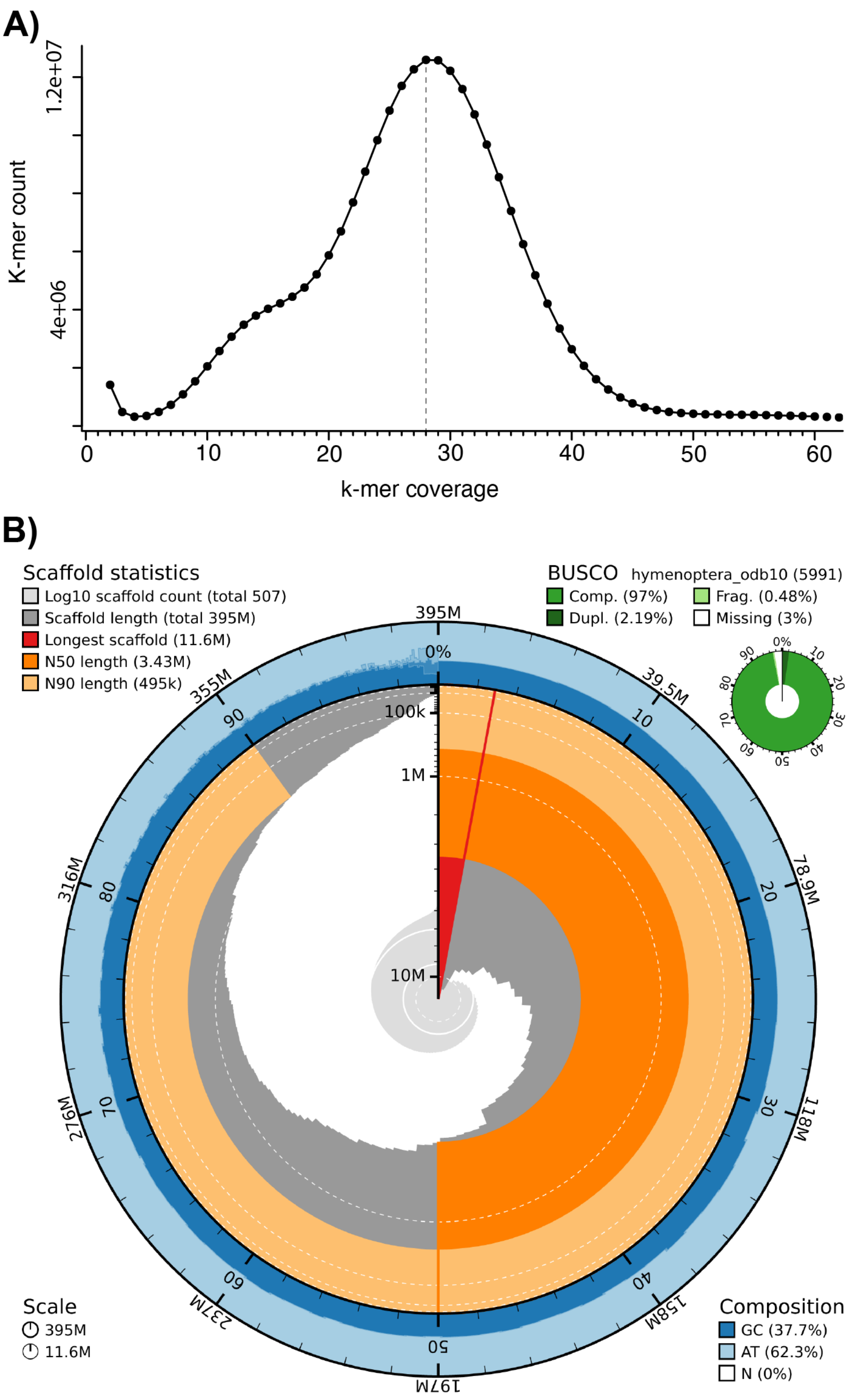

Fig. S2. Genome size estimate and primary assembly properties. A) Distribution of k-mer (k=19) counts and coverage. Dashed line indicates homozygous peak at approximately 28x coverage. **B)** Snail plot summarizing properties of the primary contig assembly, including contig length distribution and statistics, nucleotide content distribution, and completeness as estimated by the presence of universal single-copy orthologs in the Hymenoptera database (total of 5991 genes). Note that the assembly has not been scaffolded and that scaffold statistics therefore refer to contigs.

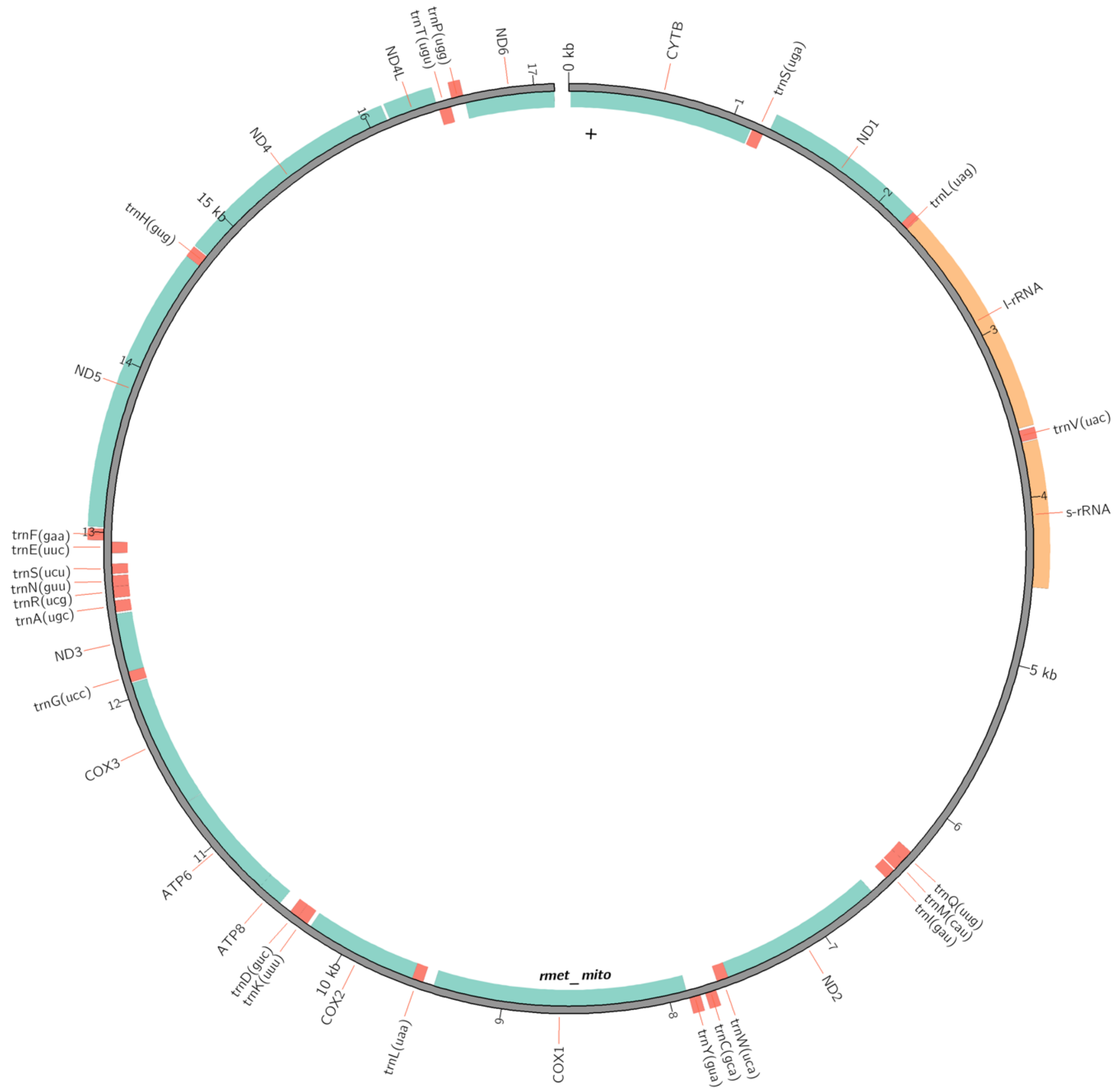

Fig. S3. ***R. metallica*** **mitogenome assembly and annotation overview.** Coloured bars indicate locations of genes, while distances are shown in kb. Genes in inner circle are located at plus strand, whereas genes in the outer circle are located in the minus strand.

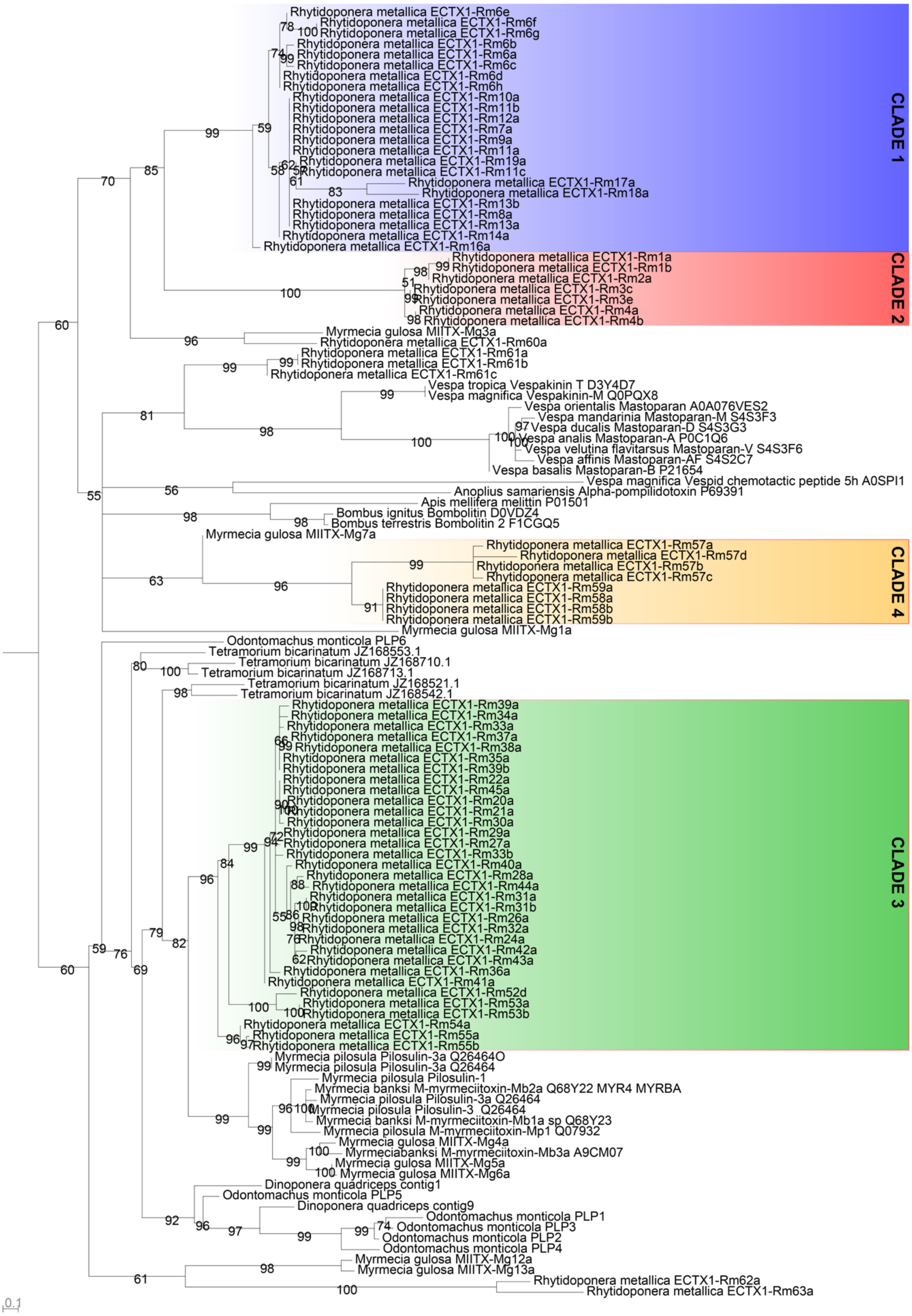

Fig. S4. Phylogeny of *R. metallica* aculeatoxins and homologues showing distribution of clades. Clades are coloured according as per Figure 1 in the main manuscript. Phylogenetic reconstruction was done by maximum likelihood under the JTT+G4 model and node support estimated by bootstrapping. Nodes with a bootstrap value less than 50% are collapsed into multifurcations.

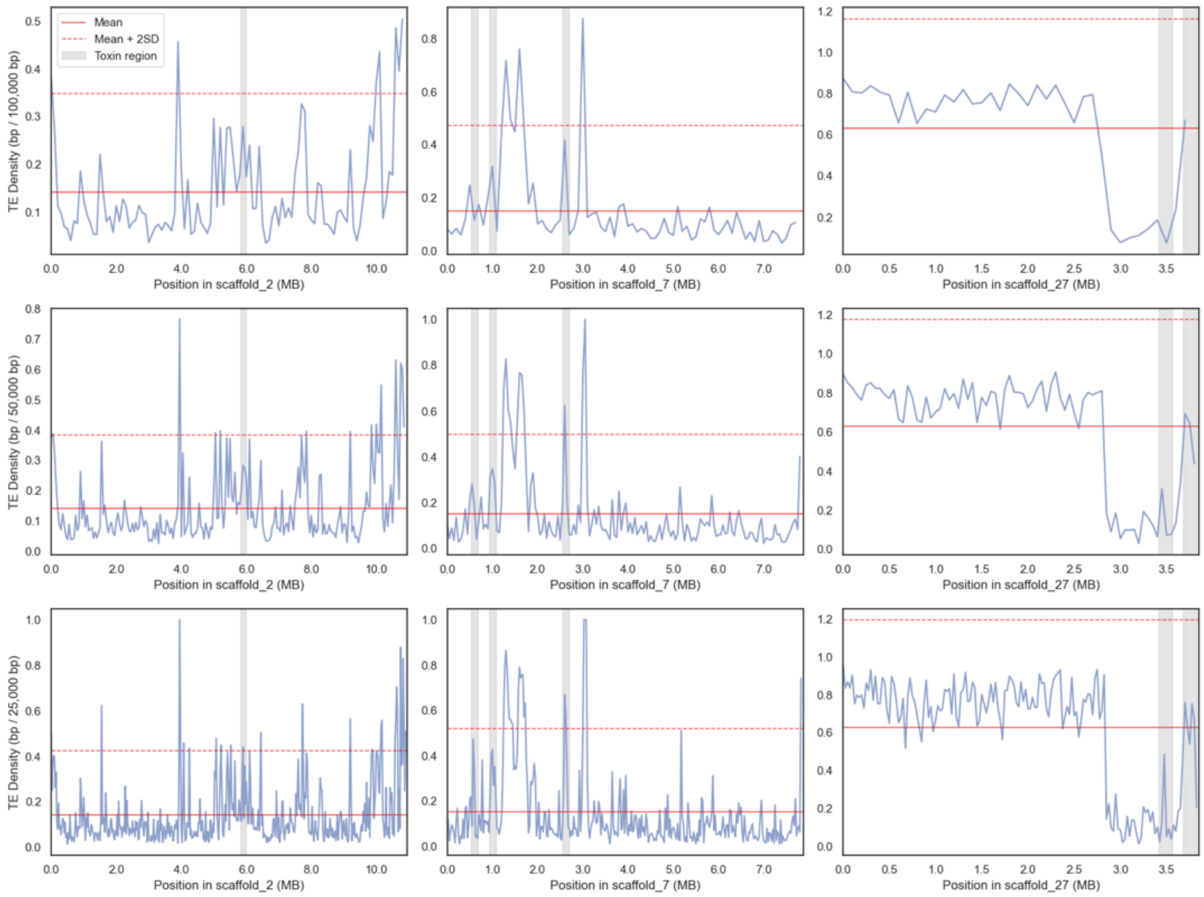

Fig. S5. Density of transposable elements in the different aculeatoxin contigs of *R. metallica.* Transposable elements present in contig 2 (left column), contig 7 (middle column), and contig 27 (right column). The different rows show TE densities for regions of 100,000 bp (upper row), 50,000 bp (middle row), and 25,000 bp (lower row). Aculeatoxin clusters are marked with grey bars. Solid red line is the contig mean. Scattered line is the contig mean plus two standard deviations.

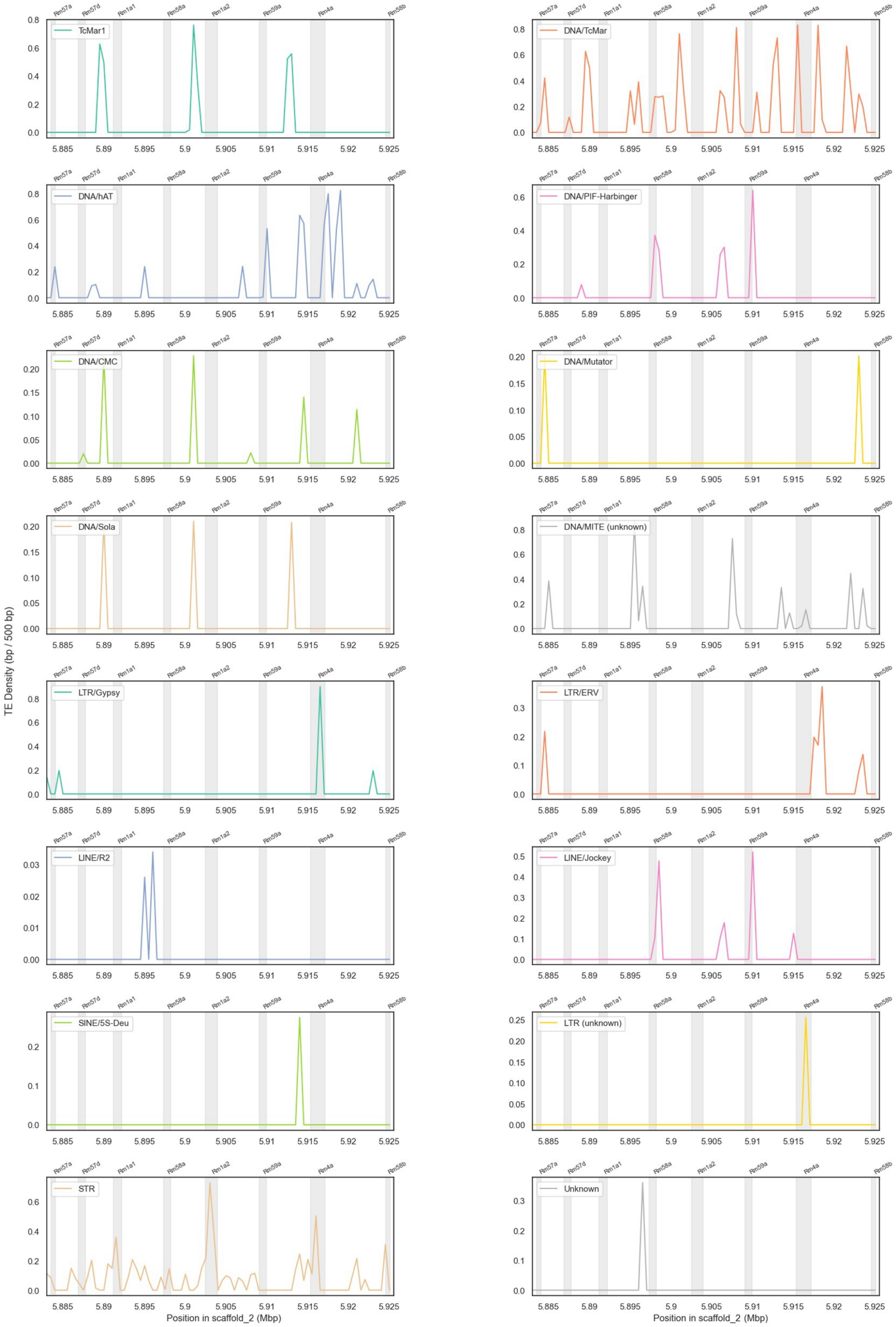

Fig. S6. Distribution of the different TEs on contig 2. For each family, density is estimated as coverage (bp) per non-overlapping sliding window of 500 bp. Each plot shows unique repeat families, coloured according to legend.

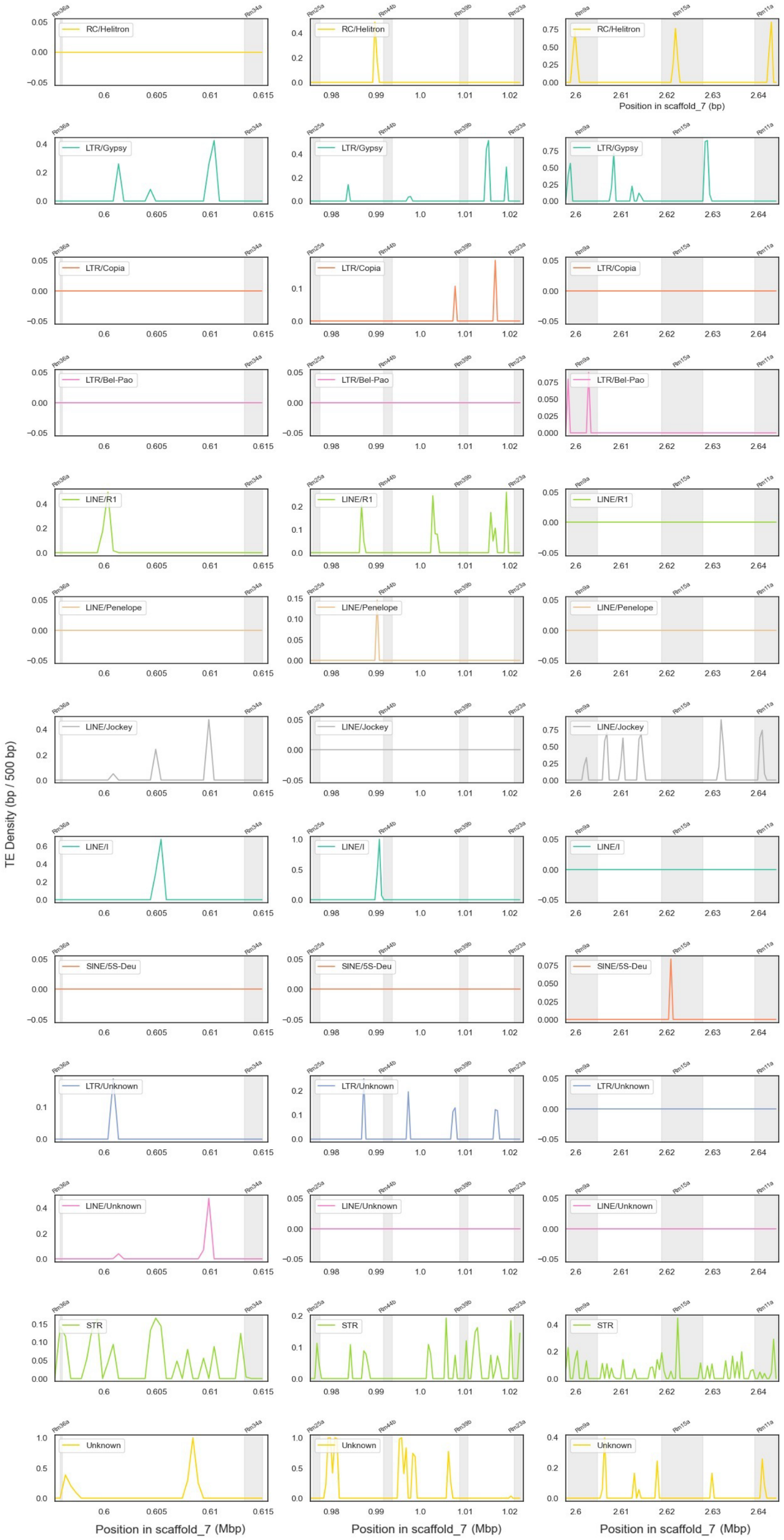

Fig. S7. Distribution of the different TEs on contig 7. For each family, density is estimated as coverage (bp) per non-overlapping sliding window of 500 bp. Each plot shows unique repeat families, coloured according to legend.

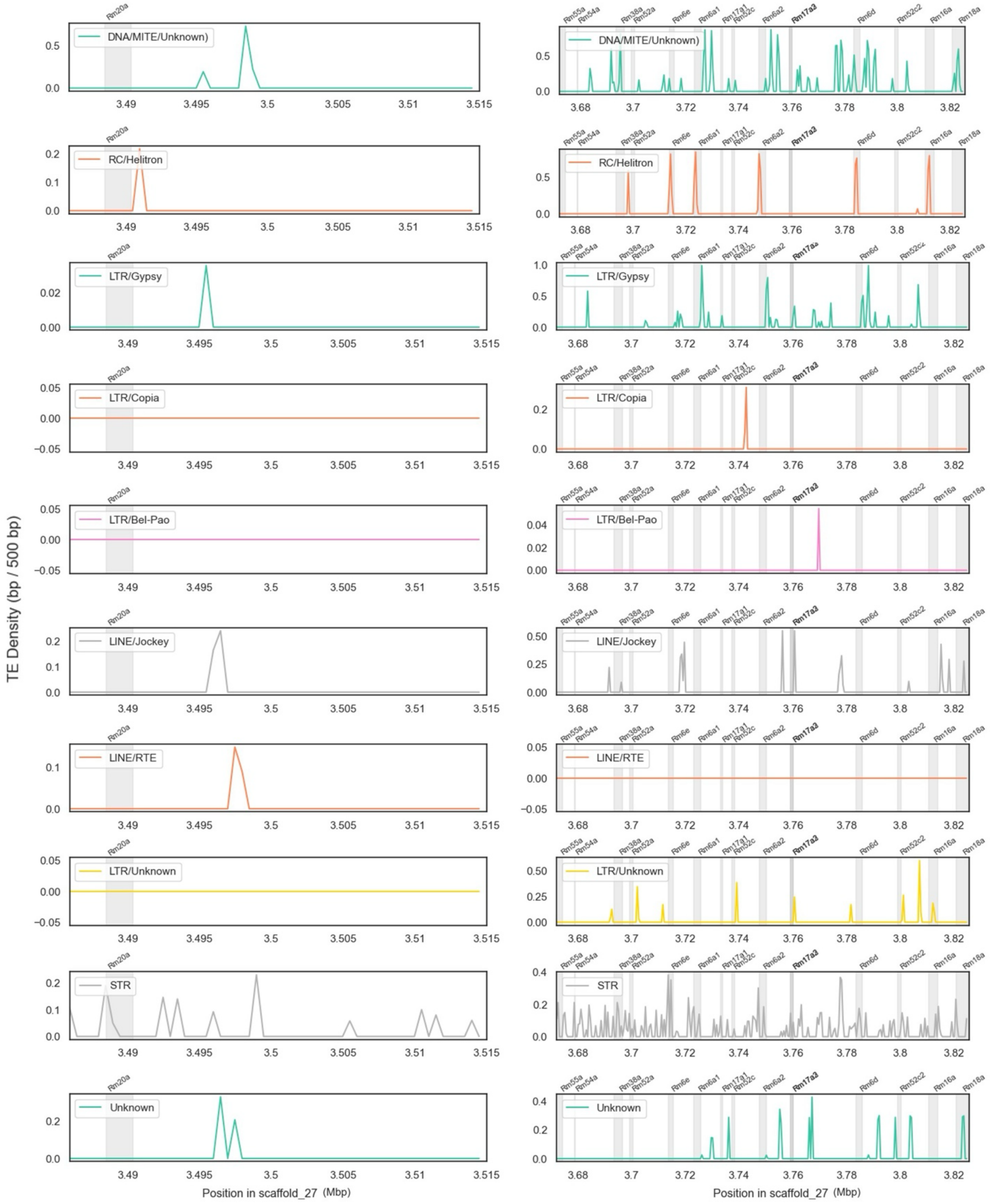

Fig. S8. Distribution of the different TEs on contig 27. For each family, density is estimated as coverage (bp) per non-overlapping sliding window of 500 bp. Each plot shows unique repeat families, coloured according to legend.

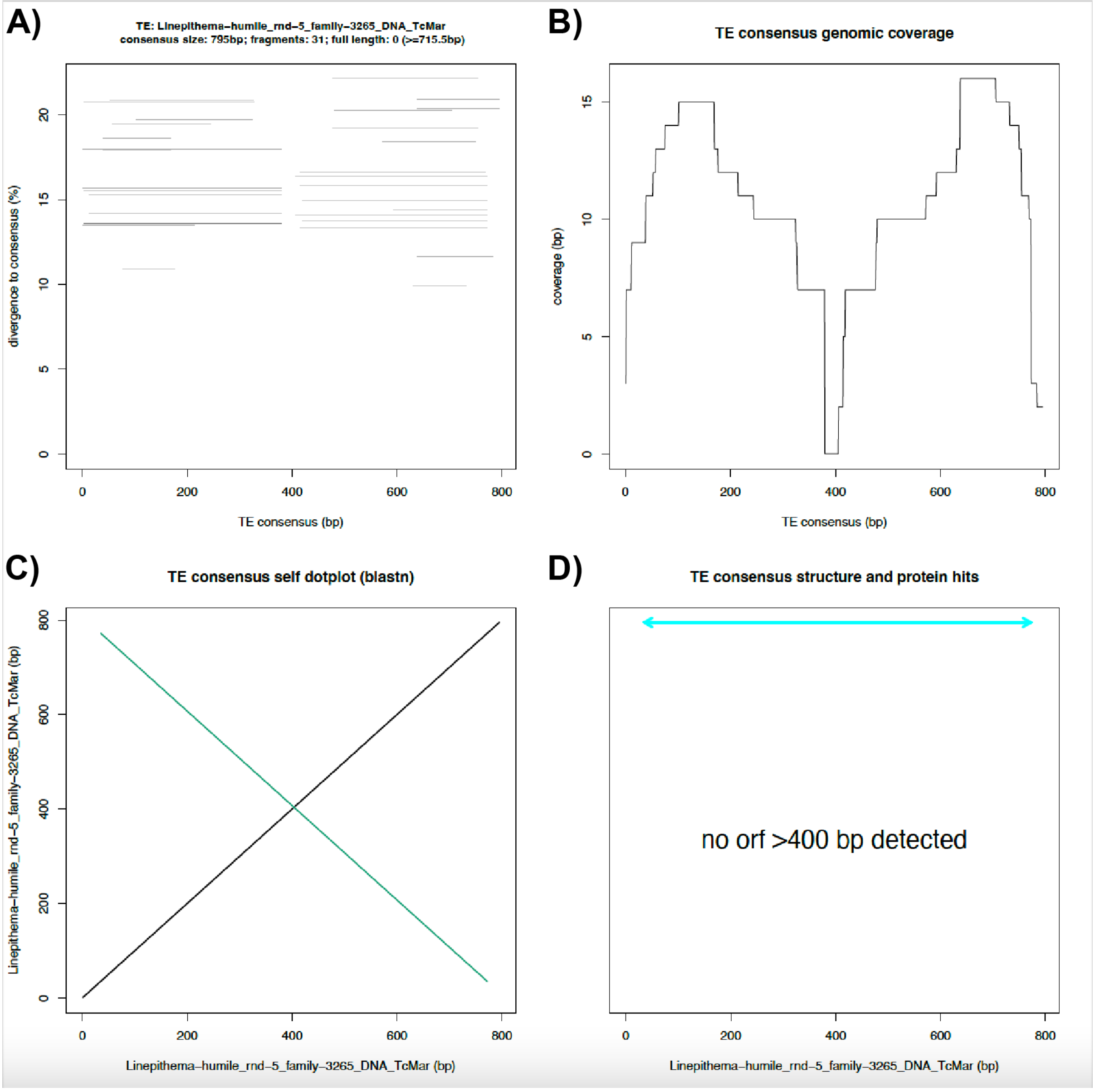

Fig. S9. Structural validation of the Tc1/Mariner-1 transposable element family. Output from TE-Aid, visualising the Tc1/Mariner-1 family residing in the aculeatoxin cluster on contig 2. **A)** Fragment and divergence plot of all Tc1/Mariner-1 insertions in the *R. metallica* genome assembly. Horizontal lines are genomic hits relative to the consensus sequence. Position on the y-axis represents divergence from the consensus sequence of Tc1/Mariner-1. **B)** Sequence coverage of genomic hits from BLAST relative to the position along the consensus sequence. **C)** Self alignment dot-plot of the consensus sequence, showing that the family is likely an old, miniature inverted repeat element. **D)** Putative ORFs and corresponding peptides located in the consensus sequence. Arrows represent micro-homologies and repetitive DNA from the self-alignment dot-plot in **(C)**.

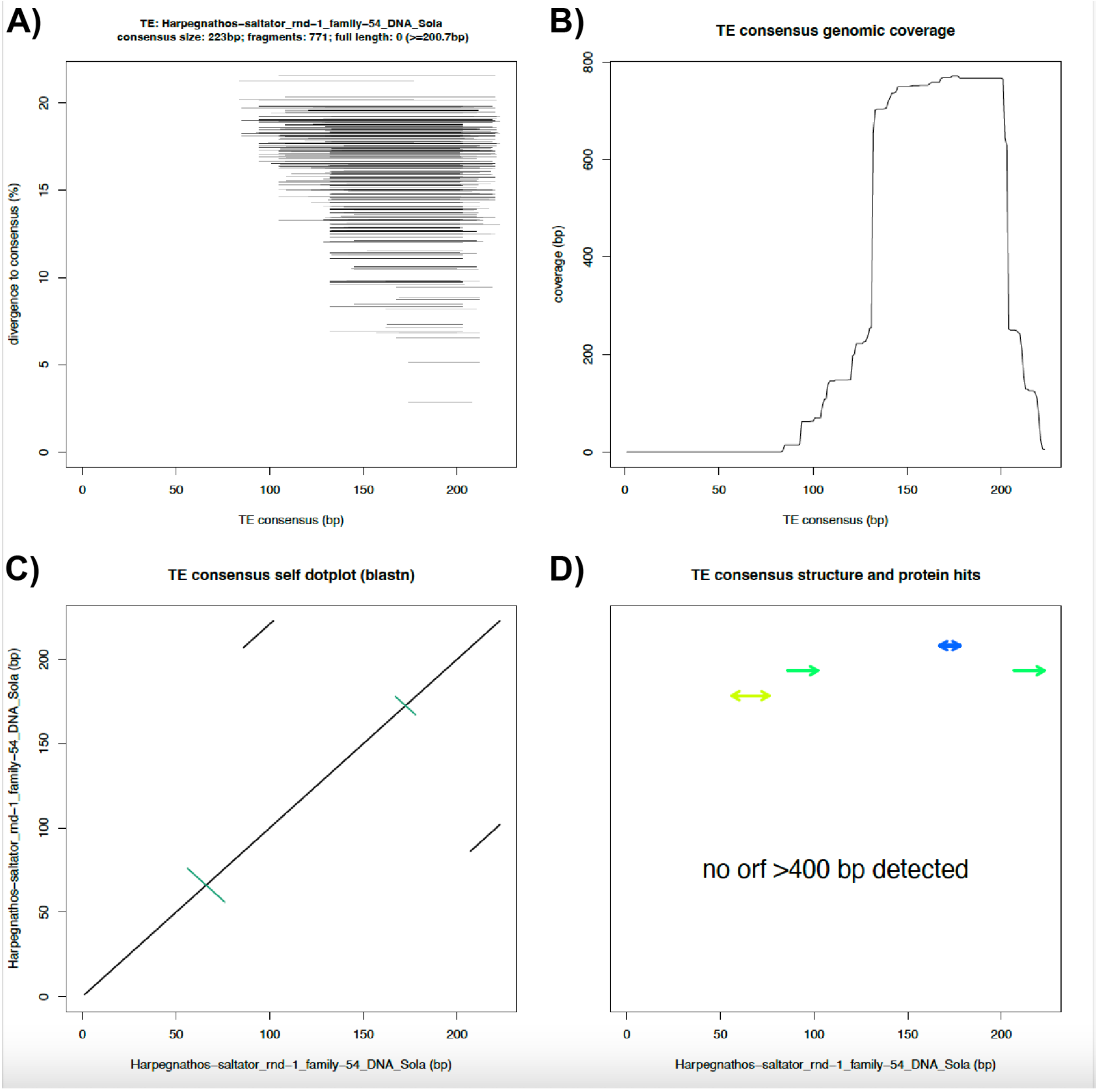

Fig. S10. Structural validation of the DNA/Sola transposable element family. Output from TE-Aid, visualising the DNA/Sola family residing in the aculeatoxin cluster on contig 2. Lack of full-sequence insertions and structural features suggests DNA/Sola is not a true transposable element. **A)** Fragment and divergence plot of all DNA/Sola insertions in the *R. metallica* genome assembly. Horizontal lines are genomic hits relative to the consensus sequence. Position on the y-axis represents divergence from the consensus sequence of Tc1/Mariner-1. **B)** Sequence coverage of genomic hits from BLAST relative to the position along the consensus sequence. **C)** Self alignment dot-plot of the consensus sequence. **D)** Putative ORFs and corresponding peptides located in the consensus sequence. Arrows represent micro-homologies and repetitive DNA from the self-alignment dot-plot in **(c)**.

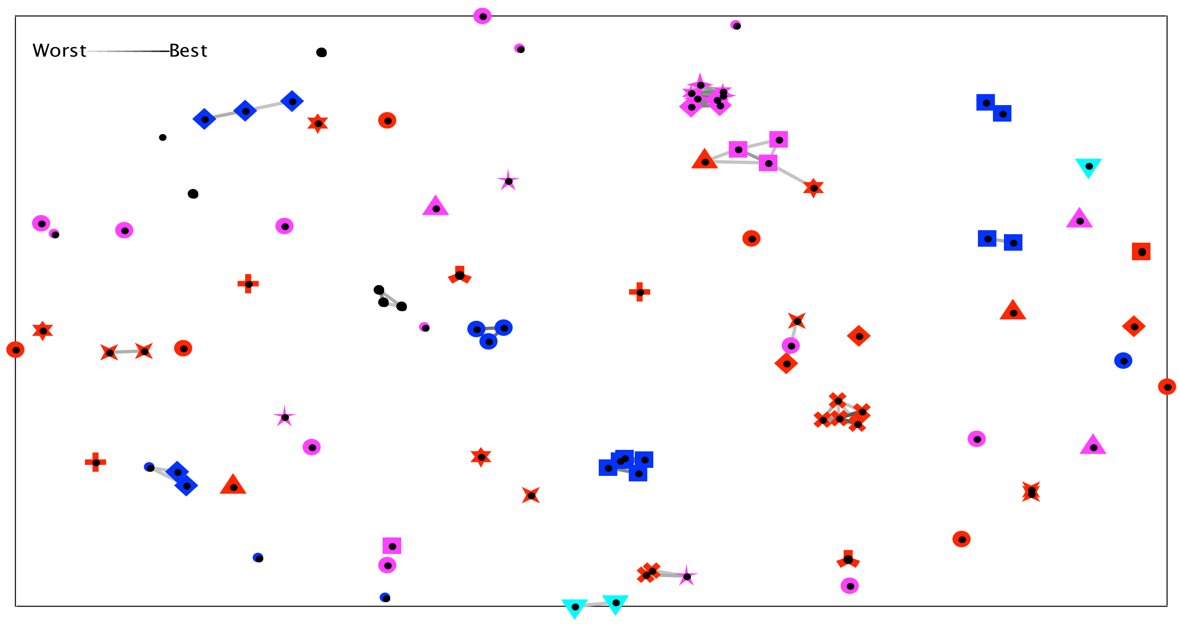

Fig. S11. Structural variation among *R. metallica* ectatotoxin paralogs and their alleles according to sequence similarity. Pairwise blastp-based clustering analysis of all mature *R. metallica* aculeatoxin amino acid sequences reveals next to no clustering of allelic variants. Colours indicate contig placement (blue=2, red=7, magenta=27, others=black), while aculeatoxins mapping to the same locus have the same symbol.

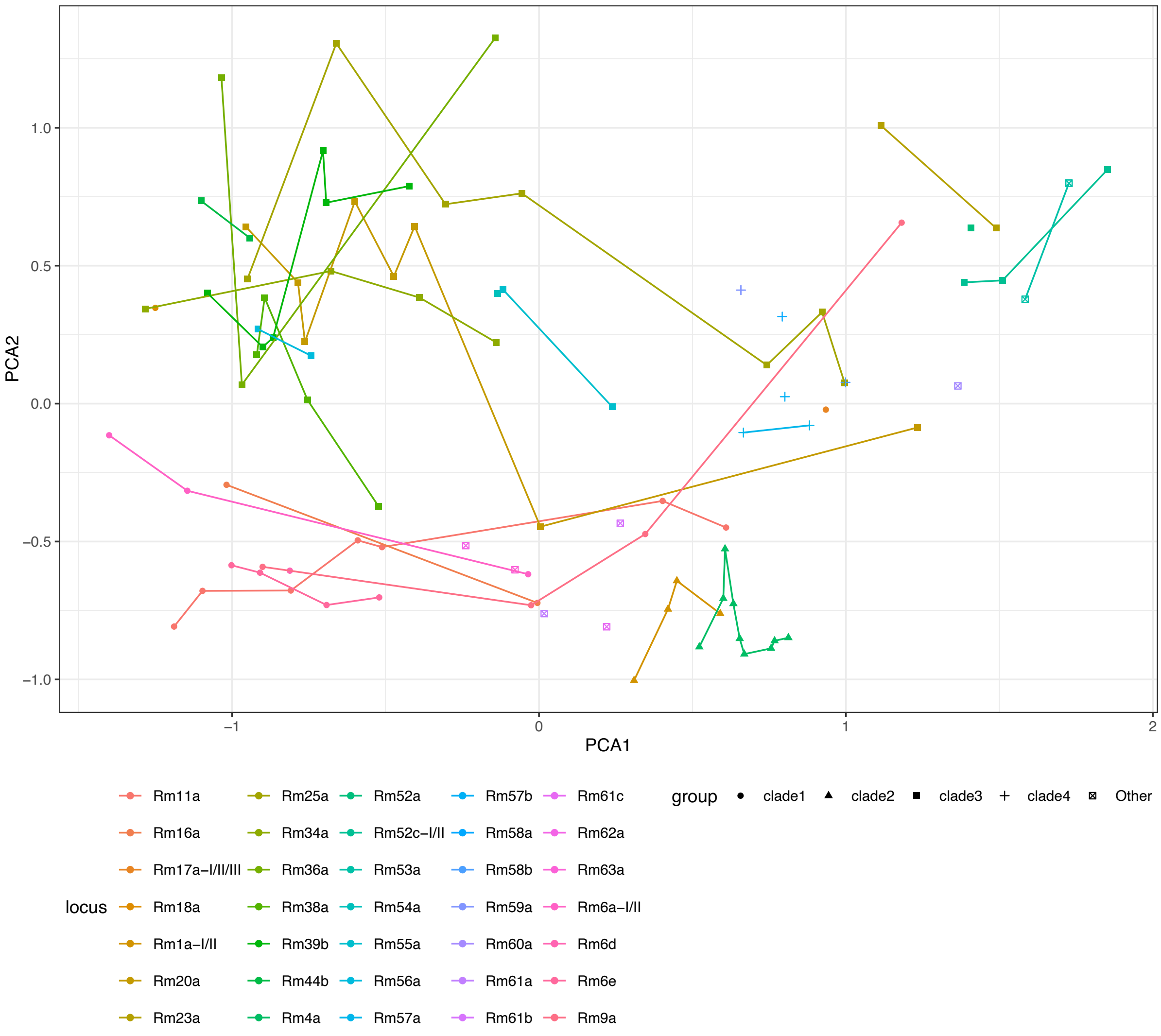

Fig. S12. Structural variation among *R. metallica* ectatotoxin paralogs and their alleles according to protein language model embeddings. Principal component analysis of projection of *R. metallica* mature ectatotoxin ProtT5 embeddings, showing the first two principal components (explained variation of 23.18% for PCA1 and 11.38% for PCA2). Allelic variants from the same locus are connected by lines, loci are shown in different colours, and members of the same ectatotoxin clade share the same symbol. While clade 3 forms a well-defined cluster, the remaining clades do not. The alleles from most loci also show large variation with extensive overlap with other loci and even clades, which indicates substantial functional diversity among allelic variants.

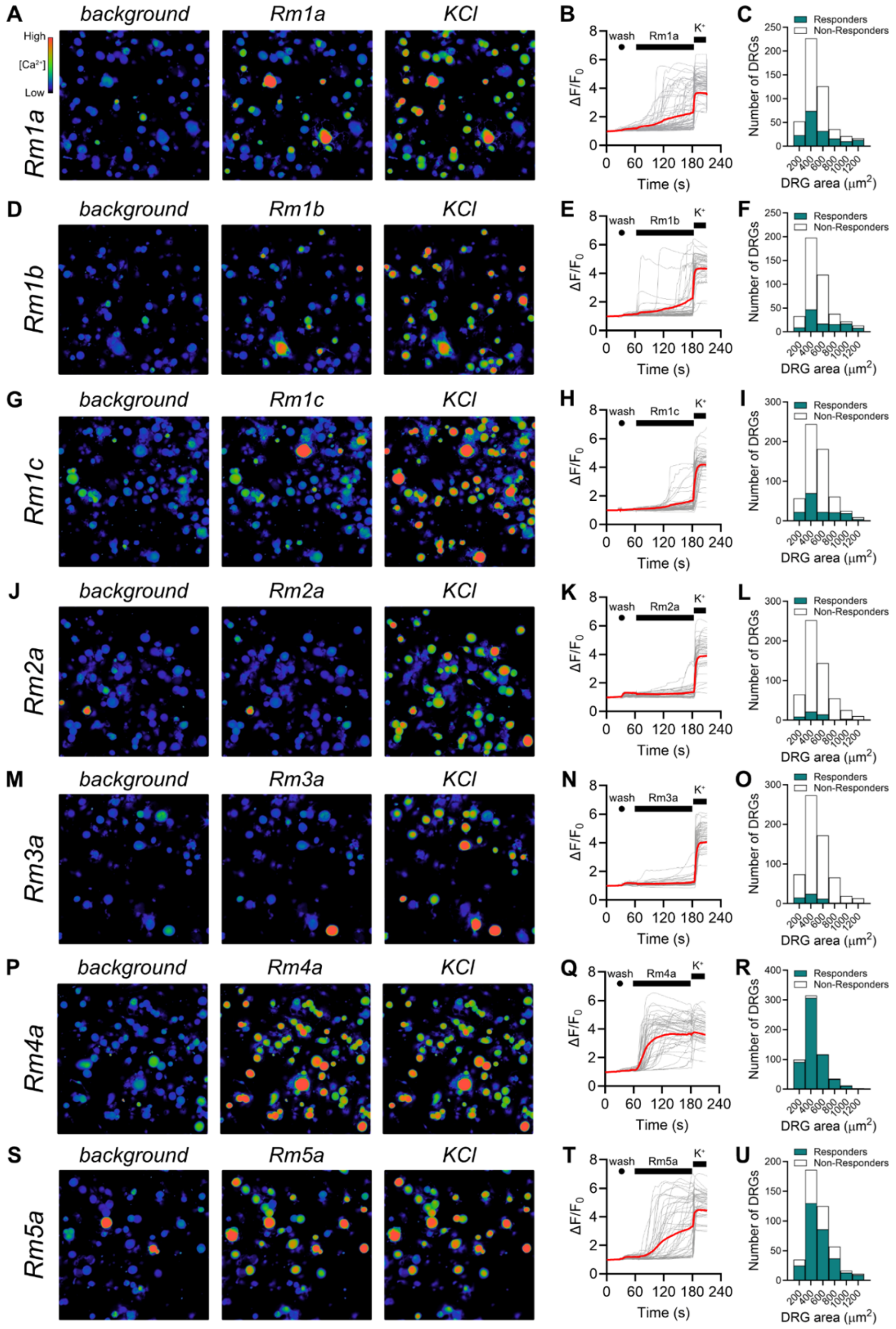

Fig. S13. Toxin allelic variants differ in activity in mouse dorsal root ganglion model of potential to cause vertebrate pain. A) Representative pseudo-colour images illustrating intracellular calcium ion concentration, [Ca2+]i in DRG neurons (left to right) before Rm1a (1 µM), after Rm1a (1 µM), and after KCl (30 mM) addition. **B)** time course of individual DRG neuron responses to Rm1a (1 µM). Each trace represents an individual neuron, and the red trace represents the average response; K+, 30 mM KCl (positive control) (representative of three independent experiments). **C)** histogram showing number of neurons that respond or do not respond (sum of three independent experiments). Each row after is the equivalent for (top to bottom) Rm1b (**D-F**), Rm1c (**G-I**), Rm2a (**J-L**), Rm3a (**M-O**), Rm4a (**P-R**) and Rm5a (**S-U**). All traces and histograms are representative of 3 independent experiments.

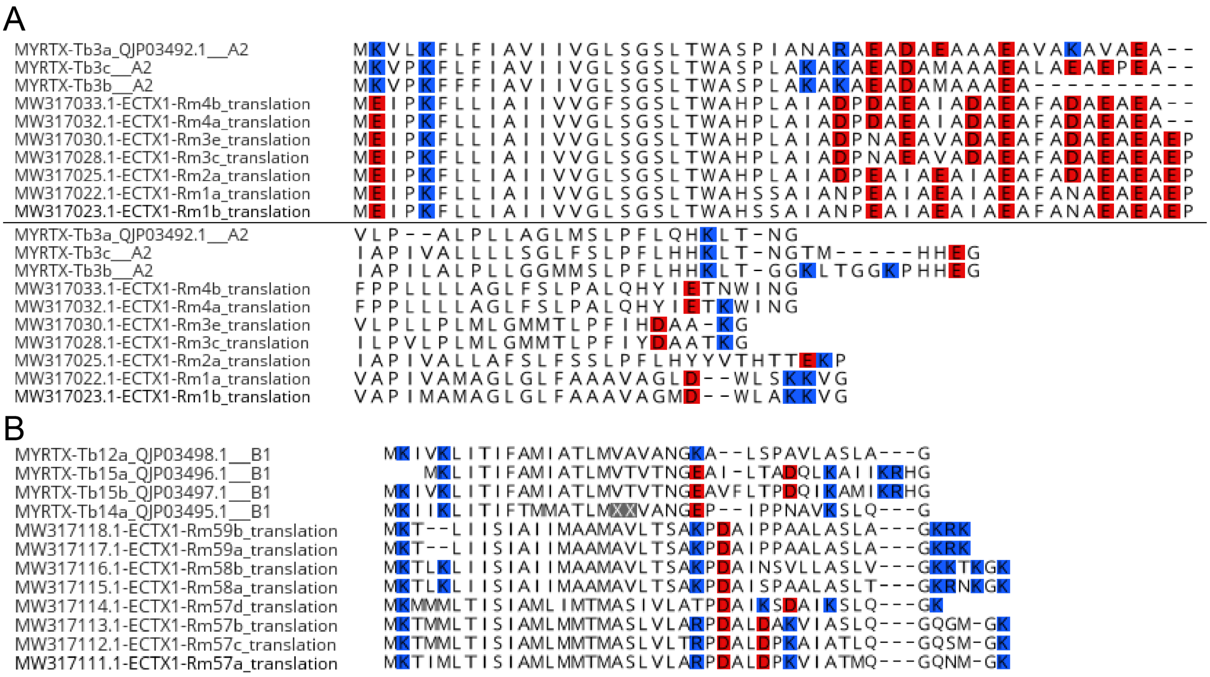

Fig. S14. Sequence alignment of putative orthologs from *R. metallica* and *T. bicarinatum*. A) Alignment of clade 2 ectatotoxins (ECTX) and subfamily A2 myrmicitoxins (MYRTX) from *R. metallica* and *T. bicarinatum*, respectively. The alignment is wrapped so that the second line starts at the mature peptide region. **B)** Alignment of ECTX clade 4 and MYRTX subfamily B1 from *R. metallica* and *T. bicarinatum*, respectively. Amino acid backgrounds are coloured according to charge, with acidic residues in red and basic residues in blue.
